# Potent PDZ-Domain PICK1 Inhibitors that Modulate Amyloid Beta-Mediated Synaptic Dysfunction

**DOI:** 10.1038/s41598-018-31680-3

**Published:** 2018-09-07

**Authors:** Edward Y. S. Lin, Laura F. Silvian, Douglas J. Marcotte, Charles C. Banos, Flora Jow, Timothy R. Chan, Robert M. Arduini, Fang Qian, Darren P. Baker, Chris Bergeron, Catherine A. Hession, Richard L. Huganir, Cassandra F. Borenstein, Istvan Enyedy, Jinming Zou, Ellen Rohde, Marion Wittmann, Gnanasambandam Kumaravel, Kenneth J. Rhodes, Robert H. Scannevin, Anthone W. Dunah, Kevin M. Guckian

**Affiliations:** 1 Biotherapeutics and Medicinal Sciences, Biogen Inc, Cambridge, Massachusetts, USA; 2 Alzheimer’s Disease and Dementia Research Unit, Biogen Inc, Cambridge, Massachusetts, USA; 3 Department of Neuroscience and Howard Hughes Medical Institute, Johns Hopkins University School of Medicine, Baltimore, MD USA

## Abstract

Protein interacting with C kinase (PICK1) is a scaffolding protein that is present in dendritic spines and interacts with a wide array of proteins through its PDZ domain. The best understood function of PICK1 is regulation of trafficking of AMPA receptors at neuronal synapses via its specific interaction with the AMPA GluA2 subunit. Disrupting the PICK1-GluA2 interaction has been shown to alter synaptic plasticity, a molecular mechanism of learning and memory. Lack of potent, selective inhibitors of the PICK1 PDZ domain has hindered efforts at exploring the PICK1-GluA2 interaction as a therapeutic target for neurological diseases. Here, we report the discovery of PICK1 small molecule inhibitors using a structure-based drug design strategy. The inhibitors stabilized surface GluA2, reduced Aβ-induced rise in intracellular calcium concentrations in cultured neurons, and blocked long term depression in brain slices. These findings demonstrate that it is possible to identify potent, selective PICK1-GluA2 inhibitors which may prove useful for treatment of neurodegenerative disorders.

## Introduction

The majority of excitatory synapses in the central nervous system are located on dendritic spines, which are specialized structures protruding from neuronal processes that function as domains for compartment-specific regulation of synaptic activity^1^. The regulation of dendritic spine density in the brain is believed to play a key role in learning and memory, and the loss of dendritic spines correlates with deficits in synaptic and cognitive functions^2,3^. Alterations in dendritic spine density can modify synaptic function and play a key role in several neurodegenerative diseases^4,5^. In Alzheimer’s disease, synapse loss, which is associated with cognitive impairment, is correlated with a reduction in dendritic spine density and elevation in soluble Aβ, and occurs prior to neuronal death^6–8^, suggesting that treatment strategies that prevent synapse loss may provide a better prognosis for Alzheimer’s disease therapy.

The AMPA-type glutamate receptor mediates the majority of fast excitatory synaptic transmission. Its trafficking into and out of the synapse regulates synaptic plasticity and dendritic spine density^9^ through interaction of the receptor subunits (GluA1-4) with specific intracellular proteins^10–12^. The C-terminus of the GluA2 subunit binds to the PDZ domain of the scaffolding PICK1 protein, an interaction that is required for AMPA receptor internalization and long term depression^13–16^. Aβ produces synaptic depression by enhancing the internalization of AMPA receptors through a GluA2-dependent mechanism resulting in a reduction in the number of dendritic spines^17^. Other reports demonstrated that soluble Aβ oligomers produced aberrant synaptic plasticity by inhibiting long term potentiation and enhancing long term depression, and also by reducing dendritic spine density^18,19^. A recent study showed that a small molecule inhibitor (BIO922, 1z in this manuscript) of the specific interactions between PICK1 and GluA2 attenuated the effects of Aβ on synapses and surface receptors^20^, suggesting that PDZ-domain mediated PICK1 interaction with the GluA2 subunit is required for Aβ effects on synapses and function.

Unlike peptides, which have limited cell permeability in the absence of a permeability tag such as a TAT fusion and undesired protein degradation, small molecule inhibitors can be designed for cell-permeability and reduced degradation. Early inhibitors of PDZ domains were short peptides which matched the key residues of the endogenous ligand^21^. Later, modified peptides, cyclic peptides and peptidomimetics, were used as tools to inhibit PDZ domains, producing limited success^21^. Recently, dimeric peptides with increased binding affinity by simultaneously interacting with multiple PDZ domains^22^ have been proposed as pharmacological tools. But none of these molecules are suitable for therapeutic intervention due to their poor potency, selectivity and/or distribution properties. Until our initial disclosure of the pharmacology of the first high affinity, non-peptide inhibitor^20^, the only reported small molecule inhibitors of PDZ domains (including FSC231 for PICK1)^23^ were weakly binding molecules. Here we describe the discovery and profiling of this series of potent and selective PICK1 inhibitors.

In this study, we report the strategic use of a high throughput screen (HTS) followed by structure based drug design in combination with an array of biochemical and cellular assays in the identification of a novel, selective, and potent series of PICK1-GluA2 PDZ inhibitors. The compounds display 200-fold better potency than the endogenous GluA2 peptide ligand, and exhibit unique pharmacological activity in stabilizing neuronal surface GluA2, functionally blocking both Aβ-induced elevation in intracellular calcium concentrations and long term potentiation in cultured neuronal models.

## Results

**Figure 1:**
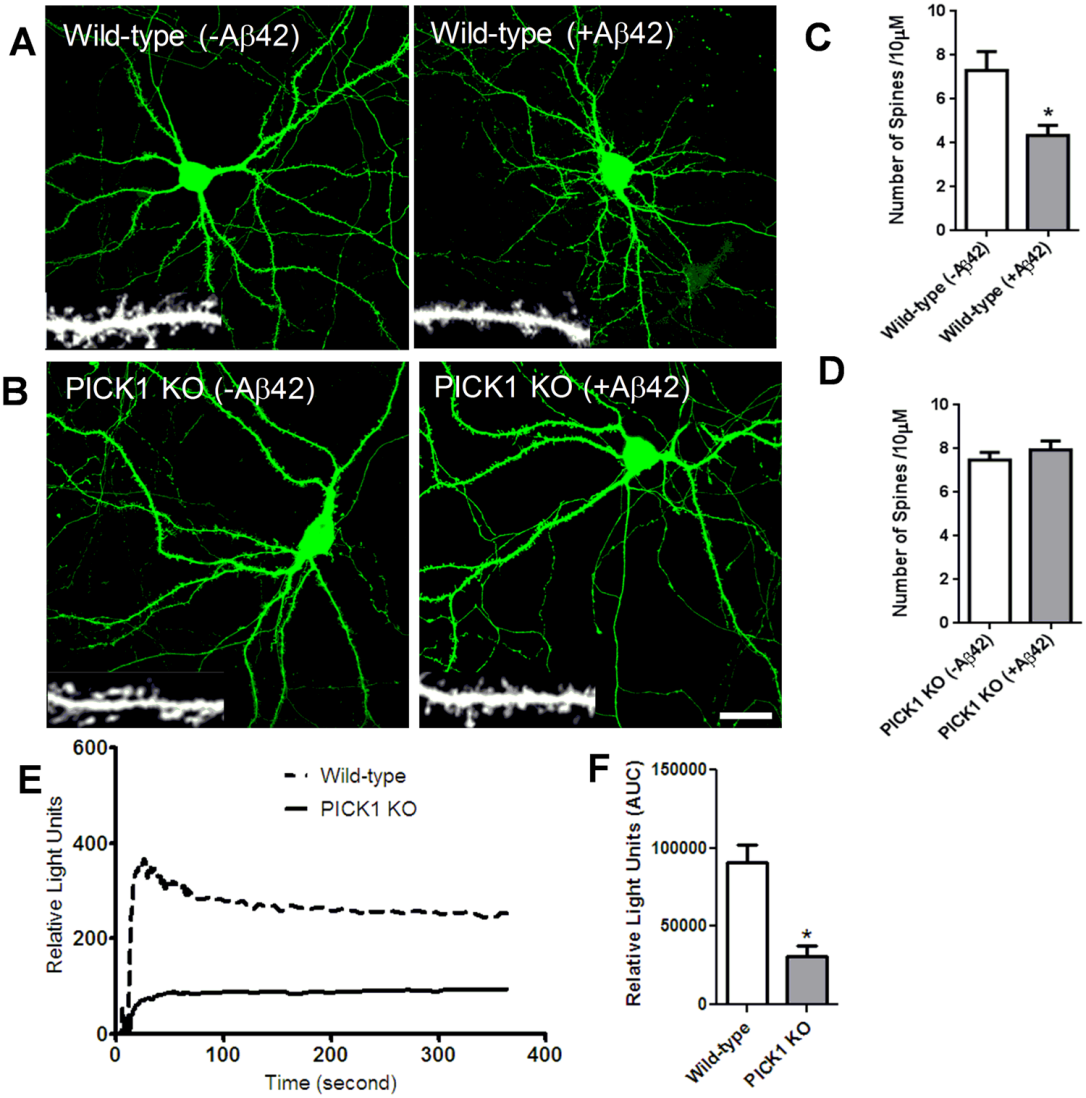
PICK1 deletion attenuates Aβ-induced modulation in dendritic spine density and intracellular calcium concentration. (**A**,**C**) Soluble oligomeric Aβ42 reduces dendritic spine density. (**A**) Cultured mouse hippocampal neurons expressing GFP to visualize neuron morphology were treated with Aβ42 (5 μM). Individual dendritic segments are shown as insets in grayscale for control (non-treated) and Aβ42-treated neurons. Scale bar, 20 μm. (**C**) Histograms show quantification of spines per 10 μm of dendrite length. n = 13 neurons for each group (*P < 0.05). (**B**,**D**) PICK1 deletion prevents Aβ42-induced decrease in dendritic spine density. Hippocampal neurons cultured from wild-type (**A**) and PICK1 knockout (KO) (**B**) mice were transfected with GFP to outline neuron morphology, and were either untreated or treated with Aβ42. Individual dendritic segments are shown as insets in gray scale for wild-type and PICK1 KO neurons. Scale bar, 8 μm. (**D**) Histograms show analysis of spines per 10 µm dendrite length in PICK1 KO mice neurons. n = 13 neurons for each group.(**E**,**F**) PICK1 attenuation decreases intracellular calcium concentration. (**E**) AMPA receptor- dependent calcium influx was measured in hippocampal neurons cultured from wild-type and PICK1 knockout (PICK1 KO) mice. (**F**) Quantification of intracellular calcium concentration in wild-type and PICK1 KO mice hippocampal neurons (*P < 0.05).

We developed a method to assess the importance of pharmacological inhibition of PICK1 on Aβ-mediated changes in synaptic morphology targeting dendritic spine density, using neurons generated from PICK1 KO mice^24^. The efficiency of deletion of PICK1 protein in cultured neurons was demonstrated by the lack of detectable protein on immunoblot (data not shown). Treatment with Aβ significantly reduced spine density in wild-type neurons compared to untreated controls (Fig. 1A,C), consistent with previous observations showing that Aβ decreases spine number in dissociated neurons^18,19^ and organotypic slice cultures^17^. In contrast, application of Aβ on neurons lacking PICK1 did not alter the density of dendritic spines (Fig. 1B,D), suggesting that PICK1 is involved in the regulation of spine integrity of neurons.

We also developed an assay to determine the functional effect of PICK1 inhibition, by measuring AMPA receptor-dependent intracellular calcium concentrations in neurons cultured from wild-type and PICK1 knockout mice using the FLIPR system as described in Methods. PICK1-deficient neurons showed significant decrease in the levels of intracellular calcium relative to wild-type neurons (Fig. 1E,F), suggesting a role for PICK1 in regulating neuronal cytosolic calcium concentrations.

**Figure 2:**
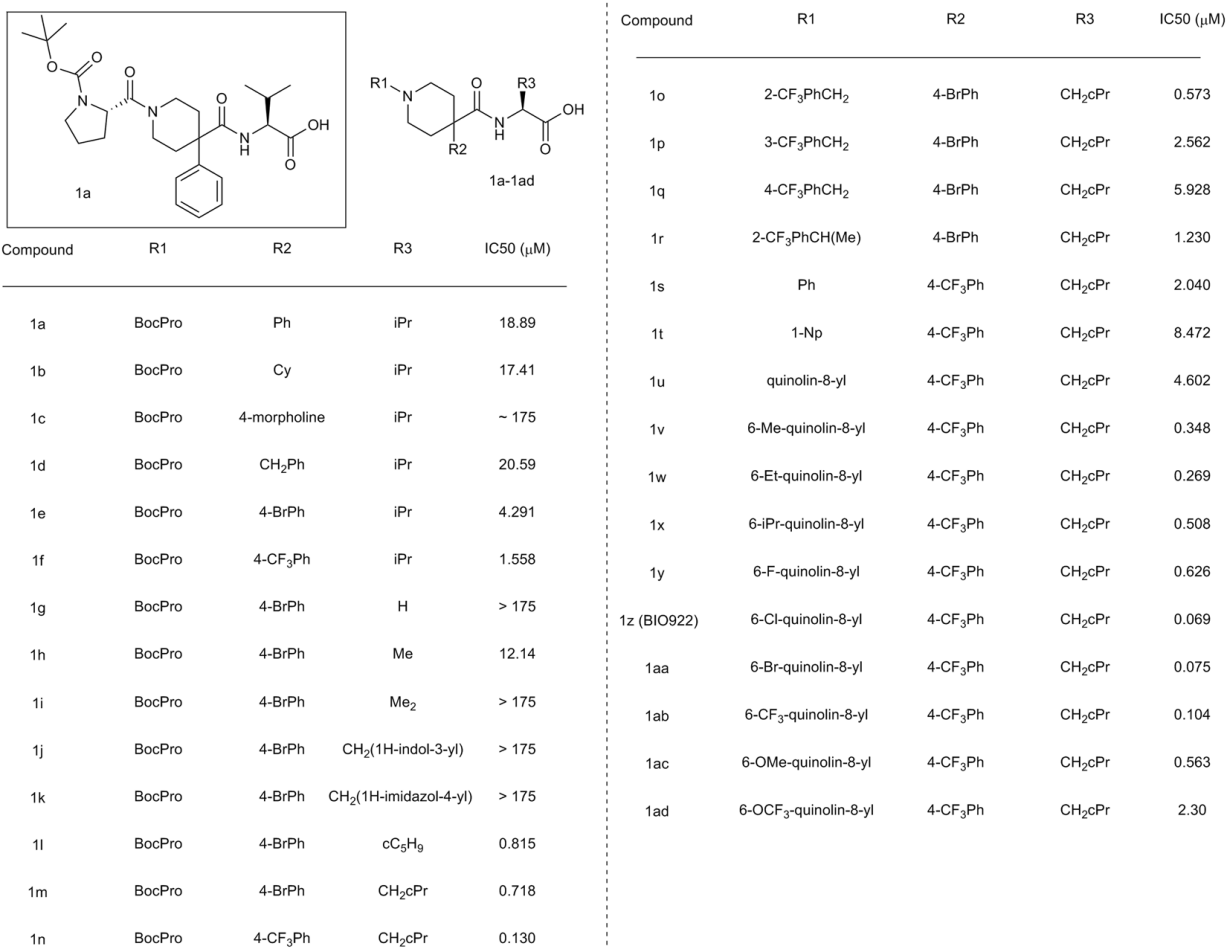
Starting point from HTS (1a) and structure-activity relationship of PICK1 inhibitors. IC50 represents value obtained using BIO424 as a tracer.

Using the array of assays, we explored the possibility of developing a potent pharmacological inhibitor that disrupted the PICK1-GluA2 peptide interaction. A high throughput screen was performed utilizing a FRET-based readout to identify compounds that prevent binding of a His-MBP PICK1 construct (amino acids 1–356) to a 9 amino acid c-terminal peptide from GluA2 that was labeled with the fluorescent fluorescein dye FITC. The screen was done with 273,000 compounds at a concentration of 50 μM (Supplemental Table 1). A 0.5% hit rate was seen when using an 18% inhibition cutoff, resulting in 1468 compounds. The Z’ for the screen was 0.75. After confirmation in triplicate, IC50s were generated on the remaining 500 compounds. In general, the hits emerging from the screen were either capped di- and tri- peptides or compounds that would be classified as frequent hitters, and only 119 of the 500 had IC50s under 50 μM. The singleton (1a, Fig. 2) had an IC50 of 19 μM and represented a non-peptide-based interesting starting point for further elaboration; we synthesized analogs to determine whether its potency could be improved by modification of the R1, R2 and R3 groups. (Fig. 2).

**Figure 3:**
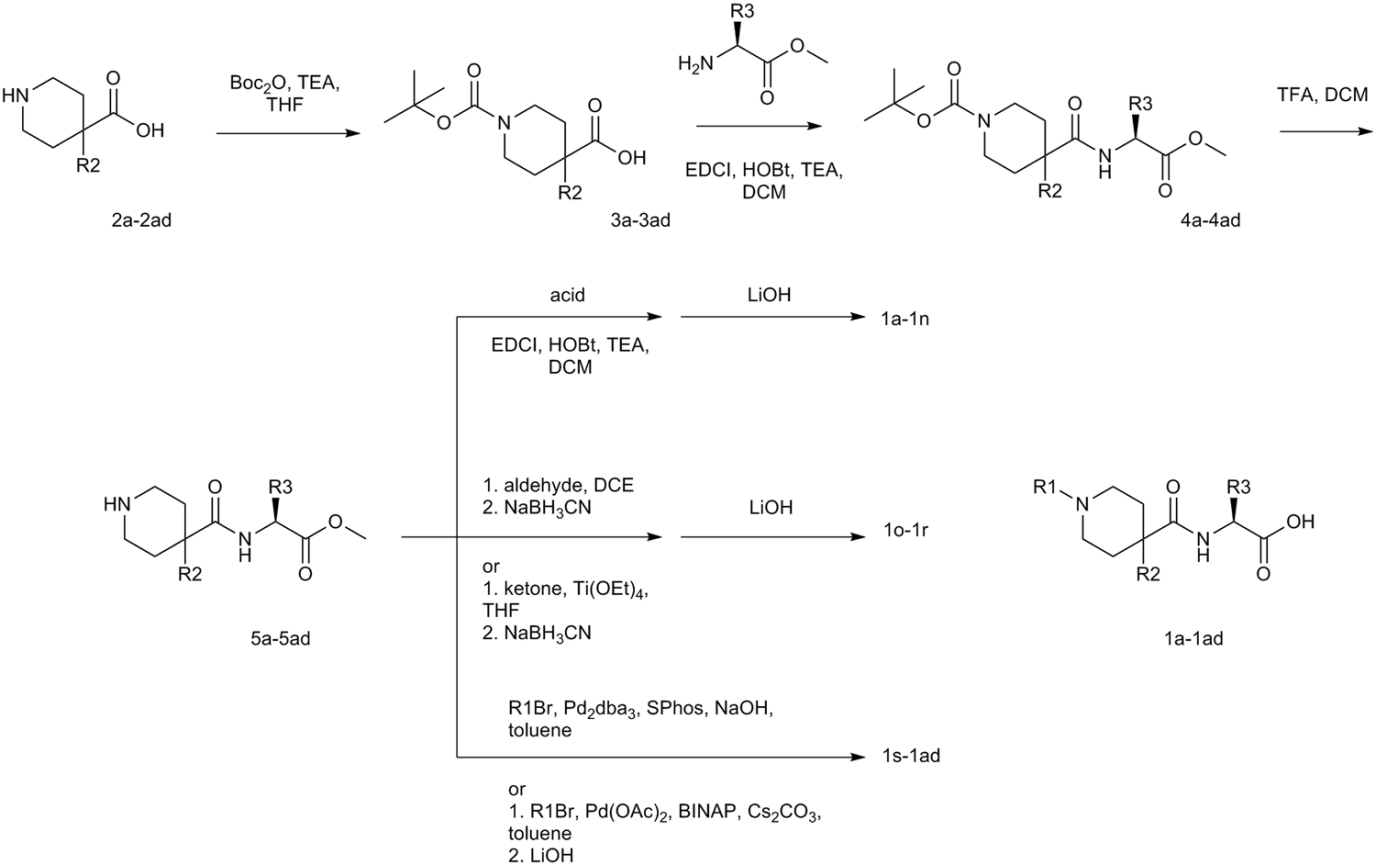
Synthetic scheme for 1a-1ad.

Synthesis of the compounds (Fig. 3) was carried out starting with 4,4- disubstituted piperdine acids (2a-2ad). The piperidine was protected with Boc and then the corresponding amino acid was coupled using standard conditions. Deprotection of Boc with TFA freed up the piperdine for amide formation (1a-n), reductive amination (1o-1r), Buchwald-Hartwig amination (1s-1ad) chemistries to give the final compounds after hydrolysis of the ester.

To determine whether the compound’s potency could be improved, we first investigated the structure-activity relationship (SAR) of the 4-phenyl ring (R2) on compound 1a (Fig. 2). When replaced with cyclohexyl (1b) or benzyl (1d), the potency was unchanged from the hit, whereas replacement with morpholine (1c) led to an approximate 10-fold loss in potency. But a significant 4-fold gain in potency was found when a bromine was added at the 4-position of the phenyl ring (1e) and this was further improved by 2–3-fold when the bromine was replaced by trifluoromethyl group (1f), resulting in a compound with a 1.6 μM IC50. Interestingly, when the CF3 phenyl substitution was moved to the 3-position of the ring, a slight gain in potency over phenyl alone was observed (data not shown), but this resulting molecule was approximately five-fold less potent than the 4-substituted CF3. Therefore, 4-substitution was kept constant and we moved to investigate the SAR of the R3 group of the molecule.

We explored replacement of the valine amino acid (R3). We first truncated to hydrogen (1g) and this resulted in a complete loss of potency. Addition of methyl as the (S)- enantiomer (1h) led to a compound with an IC50 of 12 μM, which is a 3-fold loss relative to the (S)-isopropyl base case. We evaluated the importance of chirality by synthesizing the (R)- and (S)- enantiomers of the isopropyl in another context (data not shown) and switching from (S)- to (R)- resulted in complete loss of potency illustrating the importance of chirality in improving binding affinity. Addition of a second methyl group to make the *gem*-dimethyl led to a compound that also lost all potency (1i). Further optimization of the isopropyl was explored through cyclization and the cyclopentyl (1l) was found to increase the potency by five-fold. Interestingly, a significant loss in potency was seen by moving to a smaller (cyclobutyl, ~100x) or larger (cyclohexyl, ~70x) group utilizing assay conditions where a different FRET probe was used (data not shown). The only other optimal substitution was addition of cyclopropylmethyl (1m) which also resulted in a 5-fold improvement in potency relative to the isopropyl. As with the cyclopentyl, a sharp change in SAR was seen in this region and the corresponding cyclobutylmethyl was found to be 15-fold less potent (data not shown). A further increase in potency with the cyclopropylmethyl in place was seen upon substitution of the 4-Br phenyl R2 with 4-CF3 phenyl (1n), consistent with that seen previously, which led to an additional 5-fold increase in potency to 130 nM.

In exploring the R1 position, we attempted to replace the Boc-proline (R1) with a substituted benzyl. 2-CF3 benzyl substitution (1o) resulted in a compound that was equipotent to the Boc-proline, while substitution of CF3 in the 3- and 4- positions (1p and 1q) resulted in a significant loss of potency. Introduction of methyl in the benzylic position of 1o gave 1r, which was tolerated, but did not lead to an increase in potency. Smaller and larger substituents were investigated in the 2-, 3-, and 4- positions of the benzyl and the SAR was similar to that seen with the CF3 in which the 2- position was optimal and potency decreased when the substituent was placed at the 3- or 4- positions (data not shown). In general, our initial attempts in exploring the R1 region did not result in improved inhibitor potency.

**Figure 4:**
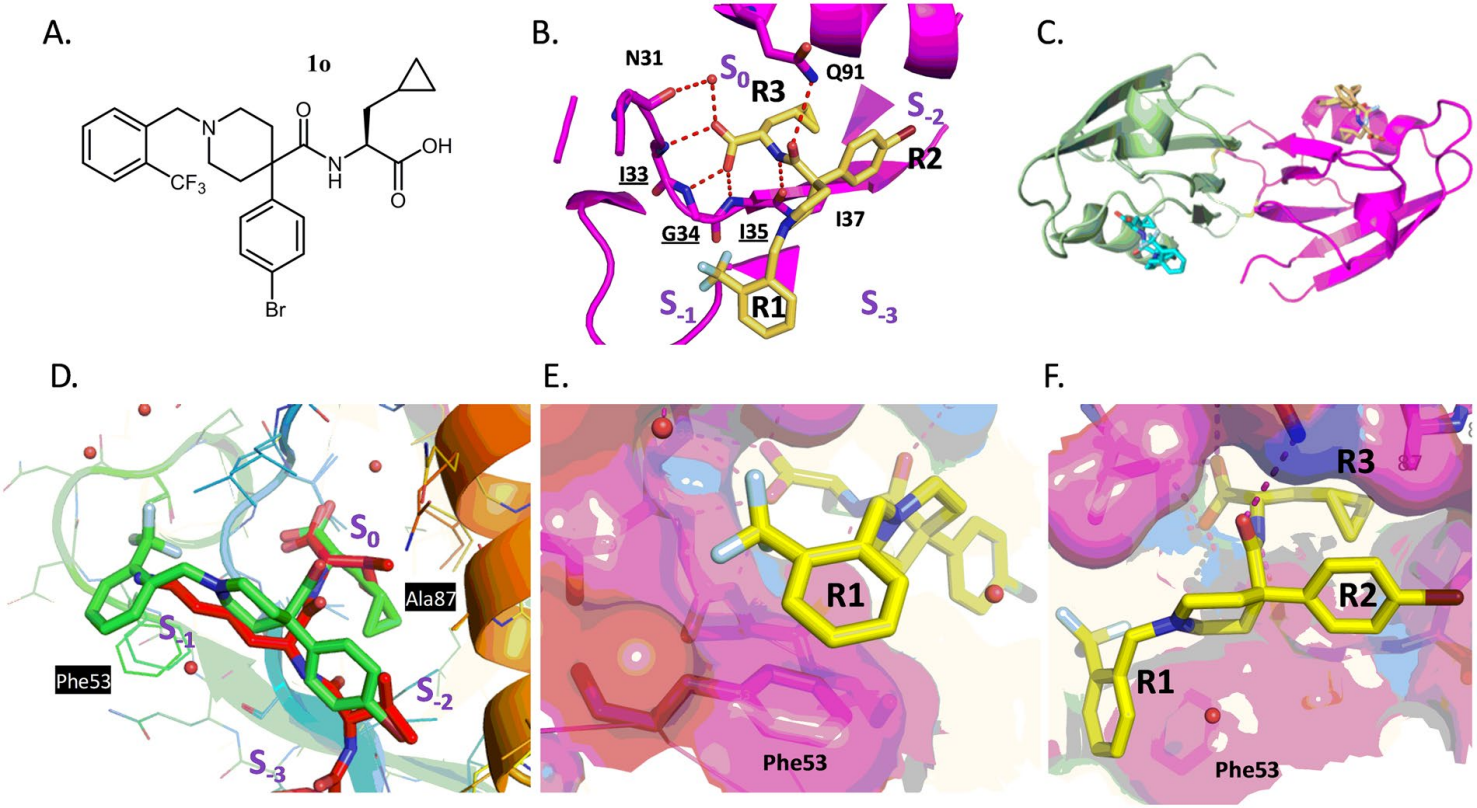
Structure of PICK1 with compound 1o. (**A**) Structure of compound 1o. (**B**) Specific binding of 1o to the PICK1 PDZ domain showing a 3-pronged pharmacophore entering pockets S_0_ (“R3”), S_−1_ (“R1”), and S_−2_ (“R2”). (**C**) Overall conformation of the PICK1 dimer and location of compound 1o binding (yellow and blue) in the peptide groove of each monomer (green and magenta). (**D**) Superposition of PICK1 alpha-carbons with the 3HPK PICK1 structure highlighting difference between peptide (SVKI; Red) and compound 1o (green) (**E**), Stacking between R1 aromatic group of compound 1o and Phe53 including the subpocket (**F**), Surface representation demonstrating interaction between fluorophenyl (R2) and Ala87, Lys83.

Our next strategy was to utilize a structure-based drug design approach and visualize how the compound was interacting with the PICK1 PDZ domain by crystal structure determination of compound 1o (Fig. 4A) with the PDZ domain of PICK1. We crystallized the complex using a method of protein oxidation and carboxypeptidase treatment of the “tail-biting” dimer in the presence of 1o in a similar way as described^25^. The structure was solved using molecular replacement under standard procedures and the resulting structure was refined to an R factor of 15.5% and an Rfree of 17.7% to 1.69 Å resolution with good geometry (Supplemental Table 2).

The overall structure of the PICK1 PDZ with compound 1o can be described as a dimer of oxidized PICK1 with two inhibitors bound in two PICK1 molecules (Fig. 4C). The density (Supplemental Fig. 1) revealed that the compound binds in a subset of the peptide binding pocket. Similar to a 3-pronged anchor, the three hydrophobic regions of the compound (R3, R2, and R1) (Fig. 4B) sit in the three peptide binding sites (S_0_, S_−2_, and S_−1_), respectively, leaving peptide pocket S_−3_ unoccupied. Compared to the binding mode of the SVKI peptide from 3HPK^26^ (Fig. 4D), the longer CF3 phenyl R1 group of compound 1o binds in the S_−1_ pocket in a similar way as the lysine side chain in 3HPK and includes a stacking interaction with Phe53. There is a space between the sidechains of Leu32, Asp28, and Thr56 which forms a small subpocket (Fig. 4E) that is partially occupied by the CF3 group. Additionally, there are van der Waals interactions between the R2 bromophenyl group and the side chain of Lys83 and Ala87 in the S_−2_ pocket (Fig. 4F). Lastly, R3 of the compound nicely mimics the c-terminal amino acid of the peptide ligand and forms a hydrogen bond network with the backbone of PICK1 (Ile33, Gly34, Ile35, Fig. 4B) while the cyclopropyl alanine sidechain of R3 nicely fills the back hydrophobic pocket of S_0_.

**Figure 5:**
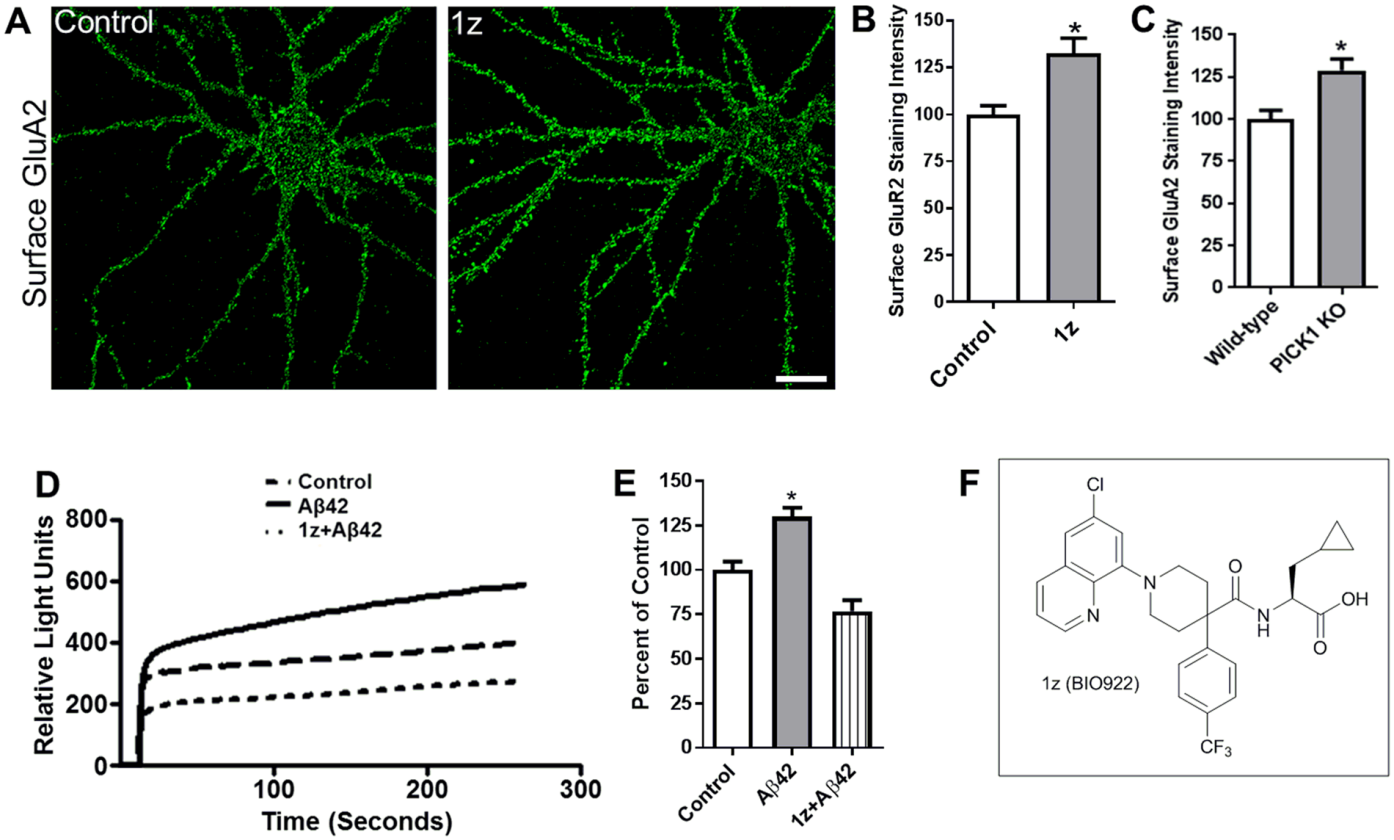
PICK1 inhibition stabilizes surface GluA2 and attenuates Aβ-mediated increase in intracellular calcium concentration. (**A**–**C**) PICK1 inhibitor stabilizes surface AMPA receptors. (**A**) Cultured hippocampal neurons were untreated (left) or treated with the PICK1 inhibitor 1z (BIO922, 3 μM) (right) for 24 hours, and immunostained for surface GluA2. Scale bar, 20 μm (**B**), Histograms show quantification of surface GluA2 immunofluorescence intensity normalized to control (non-treated) values. n = 15 control, n = 15 (1z, BIO922) treated (P < 0.005). (**C**) Histograms show quantification of staining intensities of surface GluA2 in wild-type and PICK1 KO mice neurons normalized to the wild-type group n = 16 wild-type, n = 18 PICK1 KO (*P < 0.05). (**D**,**E**) PICK1 inhibitor blocks Aβ42-induced increase in intracellular calcium concentration. (**D**) AMPA receptor-dependent calcium influx was measured in cultured hippocampal neurons left untreated (control), or treated with Aβ42 in the absence (Aβ42) or presence of PICK1 inhibitor (Aβ42 + 1z, BIO922). (**E**) Histograms show quantification of intracellular calcium concentration normalized to control (non-treated) values comparing Aβ42-treated with Aβ42 + 1z-treated cells (*P < 0.05). (**F**) Structure of compound 1z, BIO922.

The appearance of the subpocket near R1 led us to consider additional optimization ideas for the R1 position, as we observed that both the CF3 of compound 1o and the pyrrolidine ring of the potent compound 1n^25^ occupy the small subpocket described above. Docking studies suggested efficient entry into the subpocket could be enabled by introducing a *meta*-substituted aryl ring, and that a biaryl ring would optimize the stacking interaction with Phe53 when substituted directly adjacent to the ring junction (Supplemental Fig. 2). Both 1-naphthyl (1t) and quinolin-8-yl (1u) were added and showed modest potency relative to the hit 1a but much reduced potency relative to 1n. However, a significant gain in potency was seen as soon as a substitutent was added in the 6-position of the quinoline ring, consistent with our structural hypothesis. Methyl substitution (1v) boosted potency by 10-fold relative to the unsubstituted quinoline while Cl substitution (1z, BIO922) (Fig. 5F) resulted in the most potent compound in the series at 69 nM, which is 67-fold more potent than the unsubstituted quinoline and >200-fold more potent than a 9-mer c-terminal peptide of GluA2 (17.5 µM, Supplemental Fig. 3A).

Having addressed the potency issue for this tool, we then looked into how selective this compound series was for PICK1 over other closely related PDZ domains. Since the ligand GluA2 recognizes both PICK1 and GRIP we were unsure of whether we could get a selective compound. GRIP (6 PDZ domain-containing protein) is the most closely related PDZ domain to PICK1. PSD95 (2 PDZ domain-containing protein) and Shank (a single PDZ domain scaffolding molecule), are also important to counter-screen as they are also located in neurons and could be considered a liability if they were inhibited (Supplemental Fig. 3). Compound 1o demonstrated selectivity for PICK1 over PSD95, GRIP (data not shown), and Shank PDZ domains (Supplemental Fig. 3B–D). The selectivity observed for compound 1o was also observed for compound 1z^20^.

This selectivity can be explained by the co-crystal structure. A sequence alignment of the GRIP, Shank, and PSD95 PDZ domains with PICK1 demonstrates that there are differences in the residues surrounding the S_0_, S_−1_, S_−2_ pockets (Supplemental Fig. 3E). The compound selectivity can be attributed in part to the small size of the sidechain of Ala87, located between the S_0_ and S_−2_ pockets, which is unique to PICK1 (Supplemental Fig. 3E). The other PDZ domains have longer hydrophobic sidechains in that position, which would reduce the size of the S_−2_ pocket. Importantly, the selectivity may also result from interactions with the aromatic residue Phe53 (PICK1 numbering) near the S_−1_ pocket as only PICK1 has an aromatic residue in this position (Supplemental Fig. 3E). The same selectivity holds for the whole compound series; as described previously, compound 1z also showed good selectivity for the PICK1 PDZ domain^20^.

We next investigated the PK properties of compound 1z in rats to explore if it represented an appropriate *in vivo* tool compound. When dosed in suspension (CMC/Tween) at 5 mg/kg, compound 1z showed good exposure with an AUC of 2915 ng*h/mL and a Cmax of 1365 ng/mL. Not surprisingly given the properties of the compound, compound 1z showed no exposure in the brain making it unsuitable as a peripherally delivered tool compound targeting the CNS. Several other compounds within this series were tested for brain penetration and were likewise unable to cross the blood brain barrier.

Although this compound series lacks brain penetration it does have good potency and selectivity for use as a tool to investigate *in vitro* PICK1 biology. We therefore utilized two compounds 1z and 1r along with genetic tools to further explore the role of PICK1 inhibition on synaptic function.

The effect of PICK1 inhibition on stabilization of surface AMPA receptors was examined by treating cultured hippocampal neurons with compound 1z. Surface GluA2 was measured as described in the Methods. (Fig. 5A). Compound 1z increased surface GluA2 levels relative to untreated neurons (Fig. 5A,B). A similar increase in surface GluA2 was observed in neurons cultured from PICK1 KO mice (Fig. 5C). However, the total amount of GluA2 both in the cytoplasm and on the cell surface was unchanged upon inhibitor treatment (data not shown). Multiple compounds from this series were found to show a similar increase in surface GluA2 levels (Supplemental Fig. 4). These findings demonstrate that PICK1 normally functions to transport GluA2-containing AMPA receptors intracellularly, and when PICK1 is absent, the intracellular GluA2-containing receptors are released to the neuronal surface.

To determine whether PICK1 inhibition can attenuate an Aβ-mediated increase in neuronal intracellular calcium concentration, AMPA receptor-dependent calcium influx was measured in cultured hippocampal neurons treated with Aβ either in the presence or absence of compound 1z (Fig. 5D). Whereas neurons exposed to Aβ in the absence of compound 1z showed a significant increase in cytosolic calcium concentration, exposure to 1z attenuated the Aβ-induced elevation in cytosolic calcium levels (Fig. 5D,E), demonstrating that the interaction between PICK1 and GluA2 is required for Aβ-mediated elevation of intracellular calcium concentration. Two additional examples of PICK1 inhibitors from this series that reduce intracellular calcium levels upon Aβ treatment are shown in Supplemental Figs 5 and 6.

**Figure 6:**
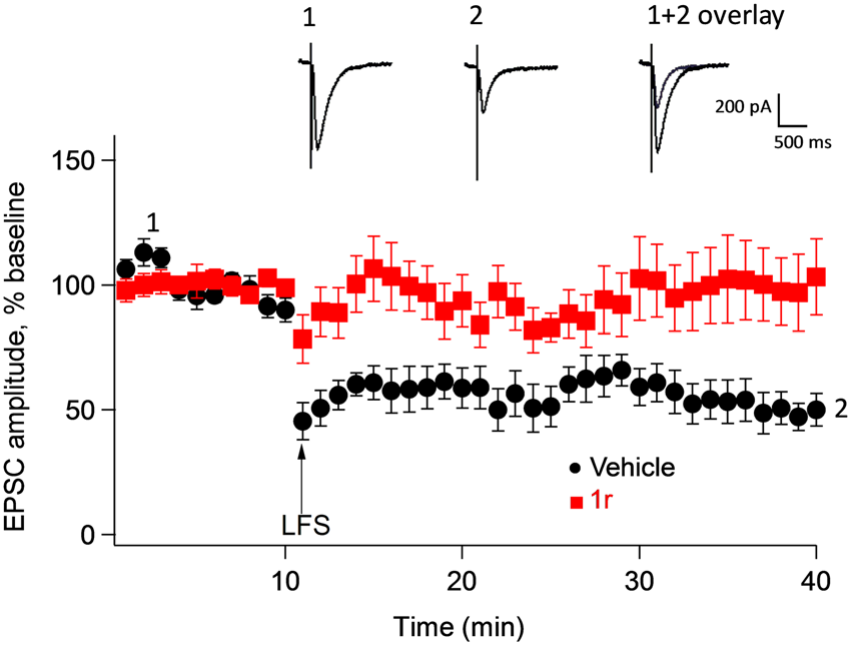
PICK1 inhibitor blocks long term depression. Long term depression (LTD) responses from vehicle-treated slices (●) and after treatment with PICK1 inhibitor (compound 1r, 4 µM) () internally before and after low frequency stimulation (LFS). Each point is the Mean ± SEM of 6 slices from 6 animals treated with control, and 9 slices from 7 animals in compound 1r. Inset shows EPSP traces of control slice before (1) and after LFS (2) revealing significant inhibition of LTD in the presence of compound 1r (1 + 2 overlay). EPSC, excitatory post synaptic current.

We further studied the role of PICK1 on synaptic plasticity by examining the effect of pharmacological inhibition of PICK1 on long term depression (LTD). Whole cell patch clamp recordings were obtained using CA1 pyramidal neurons (prepared from wild-type mice hippocampal slices) in the presence or absence of compound 1r (the solubility of which is better than compound 1z) (Fig. 6). LTD was induced and was stable for over 30 minutes in the untreated slices (Fig. 6). In contrast, LTD was not induced when compound 1r was applied to the cells (Fig. 6). These findings are consistent with evidence that PICK1 is involved in the modulation of synaptic plasticity.

## Conclusions

Recent studies, including this work, have demonstrated the role of the PICK1-GluA2 interaction in synapse structure and function, and further demonstrated its importance in Aβ-mediated modulation of synaptic density and function^20,27–30^. The emerging role of the PICK1-GluA2 interaction in synaptic biology prompted us to search for small molecule inhibitors of this protein-protein interaction to stabilize AMPA receptors on the cell surface and promote synaptic structure and function as a potential treatment for Alzheimer’s disease. Because a cell-permeable peptide would not be a suitable therapeutic, a high throughput screen and subsequent optimization of the hits by structure-based drug design resulted in a series of potent inhibitors of the PICK1-GluA2 interaction. The most potent compound in the series (1z) represents a 200-fold improvement in affinity over that of the endogenous ligand GluA2 peptide, in part by making an additional set of interactions with a phenylalanine sidechain that was not part of the ligand peptide-PDZ interaction contact set. This compound series contains the highest affinity small molecule inhibitors of PDZ domain-ligand interactions known to date. Their improved ADMET characteristics, potency and selectivity enable them to serve as useful tools for further investigation of the biology of PICK1-ligand interactions in several neurological disorders including Alzheimer’s disease^20^, Parkinson’s disease^31^, neuropathic pain^32,33^, and schizophrenia^34^. The compounds are permeable and efficacious–they attenuated Aβ-mediated spine loss and increase in intracellular calcium levels. Although the large molecular weight and acid pharmacophore limited the brain penetrance of the compounds in *in vivo* studies, this study provides scientific design principles for the development of potent small molecule PDZ domain inhibitors for other PDZ domains.

## Methods

### Dissociated primary neuron cultures

Experiments were conducted in accordance with and received approval from the Institutional Animal Care and Use Committees at Biogen Inc. The experiments were carried out in accordance with guidelines laid down by the NIH regarding the care and use of animals for experimental procedures. Primary hippocampal cultures were prepared from embryonic day 18 rodent brains as described previously^35^. Cells were plated on coverslips coated with poly-D-lysine (30 µg/mL) and laminin (2 µg/mL) at a density of 70, 000 cells per well in 12-well plates. Hippocampal neurons were grown in Neurobasal medium (Invitrogen) and supplemented with B27 (Invitrogen), 0.5 mM glutamine, and 12.5 mM glutamate, and used for the described studies. Embryonic neuronal cultures were prepared from a minimum of three independent pregnant mice on separate days in all of the studies described, and at least 13 neurons were analyzed for each experimental group.

### Preparation of amyloid beta oligomers

Synthetic Aβ42 hexafluoroisopropanol peptide was prepared to the stock concentration of 200 μM by adding 10 μL dimethylsulfoxide to 100 μg of Aβ42 hexafluoroisopropanol pellet and incubating for 30 min at room temperature with occasional mixing. Phosphate buffer was added to a final concentration of 1 mg/mL and mixed with a pipette.Using western blot we detected monomers, dimers and trimers based on molecular weight of the Amyloid beta species. The solution was kept at room temperature for 2 h and then used for experiments.

### Immunohistochemistry

Staining of surface endogenous AMPA receptors was performed in live hippocampal neurons as previously described^36^ using antibodies recognizing extracellular regions of GluA1 and GluA2. Briefly, cultured hippocampal neurons were incubated with antibody for 15 minutes at 37 °C to label surface receptors, fixed under non-permeabilizing conditions in phosphate buffer containing 2% formaldehyde/4% sucrose at room temperature, washed in phosphate buffer, and visualized with Alexa488-conjugated secondary antibody.

Staining of total AMPA receptors was done using an antibody recognizing the carboxy terminus of GluA2 in neurons fixed under permeabilizing conditions in phosphate buffer containing 2% formaldehyde/4% sucrose at room temperature, washed in phosphate buffer, and visualized with Alexa488-conjugated secondary antibody.

### Chemical synthesis and purification

See Supplementary Methods.

### Biochemical and compound selectivity assay

Fluorescence polarization (FP) and competition binding assays were used to determine PDZ binding affinities and selectivity of compounds as described in Alfonso *et al*.^20^. Briefly, a fixed concentration (5 nM) of Fluorescein isothiocyanate-labeled peptides comprising the C-terminal amino acids of GluA2 (875–883) and N-methyl-D-aspartate receptor subunit 2B (1474–1482) was used. Binding FP assays were carried out using increasing concentrations of recombinant full-length PICK1, postsynaptic density 95 (PSD95) PDZ 1–2, or Shank3 PDZ, and normalized by subtracting the tracer-only background. Competition FP assays were carried out with both fixed FITC-labeled peptides (5 nM) and non-saturating protein concentrations (PICK1, 600 nM; PSD95 PDZ 1–2, 8 µM; Shank3, 8 µM) while altering the concentrations of unlabeled peptides and 1z (BIO922) compound from 3 to 10 µM and normalized by standardizing to both binding and tracer-only controls.

### Calcium mobilization assay

Mouse hippocampal neurons from wild type E16–18 animals were isolated and cultured for 13 days *in vitro* in Neurobasal media with B27 Supplement, Glutamax, and penicillin/streptomycin (Invitrogen). Neuron cultures were then treated with Aβ in the absence or presence of PICK1 inhibitorsfor 24 hours. Calcium influx was measured using the FLIPR Calcium 4 Assay (Molecular Devices). The cells were loaded with dye for 1 hour at 37 °C and then stimulated with 40 μM s-AMPA (Sigma). Fluorescent recordings were taken using the FLIPR Tetra (Molecular Devices). Baseline measurements before AMPA stimulation were recorded and fluorescent intensity measurements were taken for roughly 3 minutes during and after stimulation. Calcium influx was calculated as the area under the curve and plotted using PRISM GraphPad software.

### Slice preparation and electrophysiology recording

Adult mice aged 8 to 9 weeks were decapitated using a guillotine and sliced at the first cervical vertebra. A cut was made through the skin in the middle of the scalp and the skull plate. The skull plate was separated and the brain was removed, and immediately submerged in iced-cold oxygenated Dissection Buffer (211 mM Sucrose, 2.6 mM KCl, 26 mM NaHCO_3_, 1.25 mM NaH_2_PO4, 5 mM MgCl_2_, 0.5 mM CaCl_2_, and 10 mM glucose) for 2 minutes. The brain was placed on an ice-cold cutting station and the brain cut along the longitudinal cerebral fissure to separate the two hemispheres. Hemispheres were placed in iced-cold oxygenated Dissection Buffer andand cerebellum was pulled away to reveal the hippocampus. The two hemispheres were arranged so that the hippocampi faced each other like two columns and the whole structure was glued onto a mounting plate, and slices were sectioned at 400 µm thickness and transferred to a holding chamber with oxygenated Recovery Buffer (124 mM NaCl, 3 mM KCl, 26 mM NaHCO_3_, 1.25 mM NaH_2_PO_4_, 2 mM MgSO_4_, 4 mM CaCl_2_, 10 mM glucose, and 1 mM kynurenic acid). Slices were allowed to recover for 1–1.5 h at room temperature before electrophysiology experiments. Whole-Cell current-clamp measurements on hippocampal CA1 pyramidal neurons were carried out at 32 to 33 °C. The cells were held at −60 mV potential and schaffer collateral fibers were stimulated. The external solution was oxygenated Artificial CSF solution (124 mM NaCl, 3 mM KCl, 26 mM NaHCO_3_, 1.25 mM NaH_2_PO_4_, 2 mM MgSO_4_, 2 mM CaCl_2_, 10 mM glucose, and 100 µM picrotoxin). The internal solution was composed of 130 mM CsMeSO_3_, 20 mM CsCl, 3 mM NaGTP, 2 mM MgATP, and 4 mM QX-314. Patch pipettes were pulled from Borosilicate glass (TW150-4, World Precision Instruments, Inc) using Pipette Puller PIP 6 (HEKA Instrument, Inc, US), and had resistance between 4 to 5 MΩ. An EPC10 amplifier with the acquisition program Patchmaster (HEKA Instrument, Inc, USA) was used for data acquisition and Igor Pro (WaveMetrics, Inc., Lake Oswego, OR, USA) was used for data analysis. An S88 stimulator (Grass, W. Warwick, RI, US) was used for stimulation. All data were discarded if series resistances varied by >30%.

### Measurement of dendritic spine density

To outline the morphology of dendrites, hippocampal neurons were transfected with green fluorescent protein (GFP). Spines were defined as protrusions of 1–4 µm in length that showed a clear ‘head’. The dendrites containing spines were manually traced and spines counted by computer and normalized by area to compute the density. Morphometric quantification of acquired images was blinded. Aβ was used at 5 µM concentration in our experiment because it did not affect neuronal viability in a lactate dehydrogenase cell toxicity assay, and also produced a statistically significant effect on dendritic spine density in a dose-response studies.

### Image analysis and data quantification

Confocal images of immunostained neurons were obtained using a confocal microscope objective (LSM 710, Zeiss) with sequential acquisition settings at a resolution of 1024 × 1024 pixels. Each image was a z-series of eight to 10 spaced at intervals of about 0.5 μm, and the resultant stack was ‘flattened’ into a single image using a maximum projection. The confocal microscope settings were kept the same for all scans. All analysis and quantifications were carried out using MetaMorph image analysis software (Universal Imaging Corporation). Dendrites from experimental groups were randomly selected and carefully traced, and the average intensity of fluorescence staining was determined for the traced regions. Intensity measurements are expressed in arbitrary units of fluorescence per square area. Acquisition and quantification of images was blinded. All data were reported as Mean ± SEM. Statistical analysis used either Student’s *t*-test or analysis of variance (ANOVA) by Bonferroni’s *post hoc* correction, with P < 0.05 considered significant.

### Cloning, expression, and purification

Two recombinant PICK1 constructs were used in this study. The PICK1 PDZ domain with an additional C-terminal fusion (NKLQQSAV) was produced for crystallography in *E*. *coli* and purified as a dimer^25^. A truncated, dimeric form of PICK1 lacking the last 59 residues was expressed in baculovirus and purified to homogeneity for use in the *in vitro* displacement assay. In general, both proteins were purified by Nickel chromatograpy followed by size exclusion chromatography. The details of the expression, purification, and characterization are described in Supplemental Methods. The following specificity targets were cloned, expressed, and purified with comparable procedures:

PSD95 protein (domains 1–2 in tandem; aminoacids 61–249) cloned with an 8XHis-TEV tag was expressed and purified from *E*. *coli*. GluN2B peptide sequence: KLSSIESDV.

Human Shank3 PDZ domain (residues 663–757) was expressed and purified from *E*. *coli* using a comparable system. GKAP peptide sequence: YIPEAQTRL.

### Structure determination

X-ray diffraction data for crystals of dimeric bCPA-treated PICK1 PDZ/1o was collected at ID-31 at the Argonne Photon Source and was processed with HKL2000. The crystals belonged to a P3_2_ space group with one disulfide bridged PICK1 PDZ/1o dimer per asymmetric unit (Supplemental Table 2). The structure was solved with MOLREP using the PICK1 PDZ-QSAV structure with the AV removed as the search model. The structure went through multiple rounds of refinement and model building using Refmac5 and Coot which led to a model with R_cryst_ of 15.5% and R_free_ of 17.7% to 1.69 Å with good geometry. The density for the compound is clear (Supplemental Fig. 2) and the structure has been deposited in the RCSB database as 6AR4.

## Electronic supplementary material

Supplementary material
