# Supplementary material for: Potent PDZ-Domain PICK1 Inhibitors that Modulate Amyloid Beta-Mediated Synaptic Dysfunction
Source: Sci Rep. 2018 Sep 7;8:13438. doi: 10.1038/s41598-018-31680-3 (PMC6128908; doi:10.1038/s41598-018-31680-3)
Supplement: Supplementary file 1 — Supplementary material [file 41598_2018_31680_MOESM1_ESM.pdf]

Supplementary Information:

Potent PDZ-Domain PICK1 Inhibitors that Modulate Amyloid Beta-Mediated Synaptic Dysfunction

Edward Y. S. Lin[1], Laura F. Silvian[1], Douglas J. Marcotte[1], Charles C. Banos[2], Flora Jow[2], Timothy R. Chan[1], Robert M. Arduini[1], Fang Qian[1], Darren P. Baker[1], Chris Bergeron[1], Catherine A. Hession[1], Richard L. Huganir[3], Cassandra F. Borenstein[1], Istvan Enyedy[1], Jinming Zou[1], Ellen Rohde[1], Marion Wittman[2], Gnanasambandam Kumaravel[1], Kenneth J. Rhodes[2], Robert H. Scannevin[2], Anthone W. Dunah[2], Kevin M. Guckian[1*]

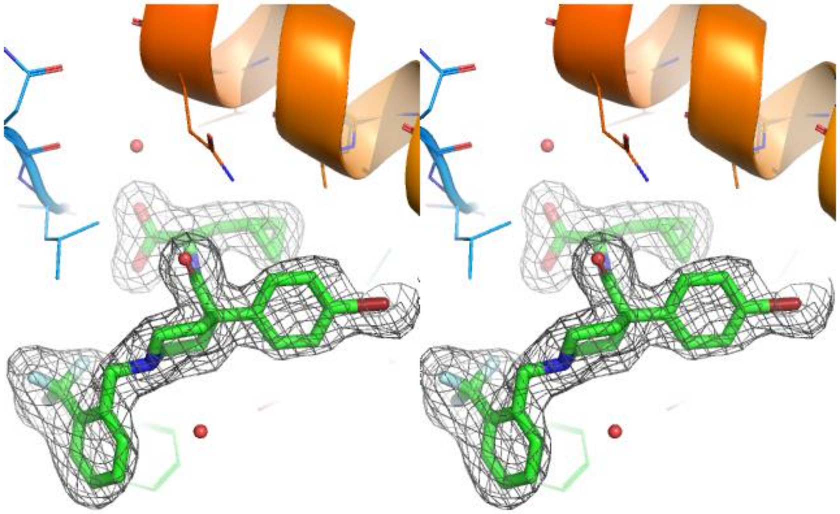

Supplemental Figure 1. Stereo view of 2Fo-Fc density for compound 1o, contoured at 1 sigma.

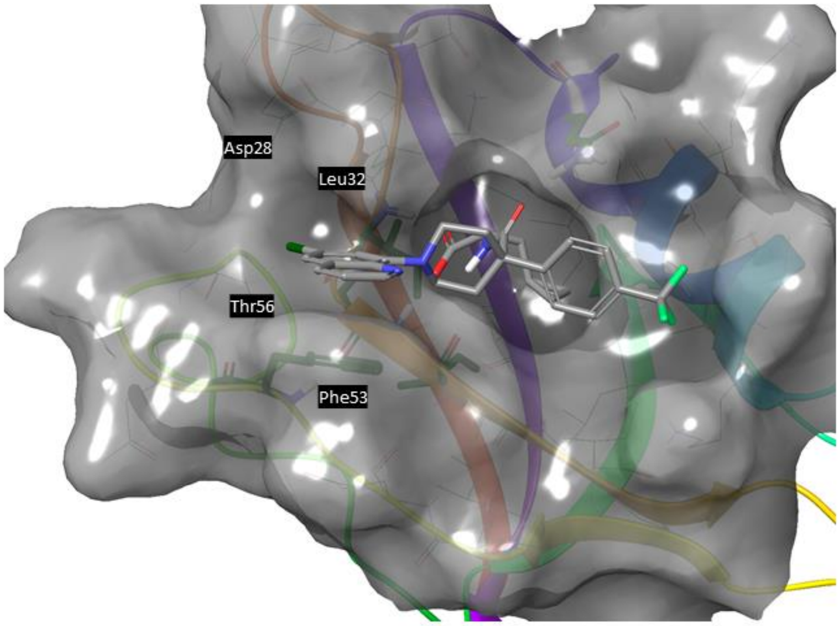

Supplemental Figure 2. Docking of PICK1 inhibitor 1z demonstrating interaction of Chloroquinoline with the S_{-1} subpocket.

Utilizing the co-crystal structure of compound 1o with PICK1, a grid was created for docking in Maestro. Compound 1z was then docked into the grid. The docking pose shows a stacking interaction of the quinoline with Phe53 and the chlorine atom inserts into a small pocket (defined by Asp28, Leu32, and Thr56) consistent with the potency increase seen upon substitution at this position.

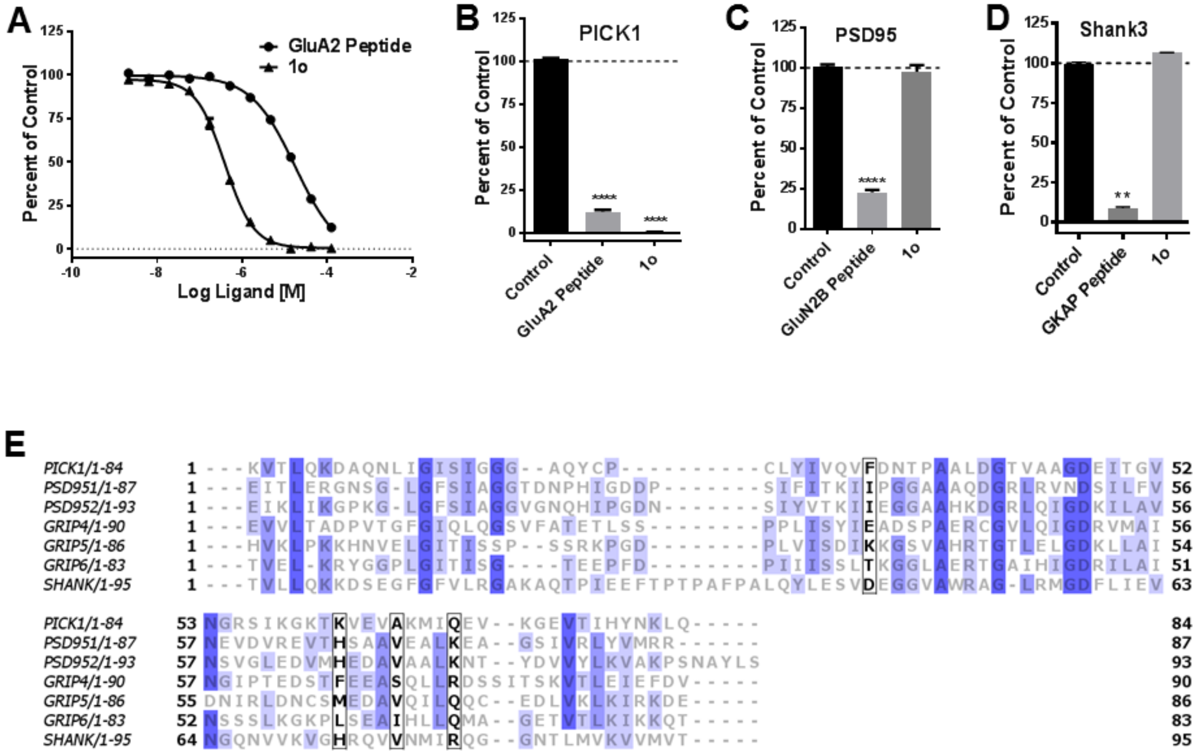

Supplemental Figure 3. PICK1 inhibitors selectively block the binding of GluA2 to PICK1 relative to other PDZ domain-containing proteins.

A, Fluorescence polarization competition binding of PICK1 inhibitor (1o) and GluA2 peptide with FITC-labeled GluA2 peptide ligand tracer. IC50 of GluA2 peptide = 17.5 μM.

B, Fluorescence polarization competition assay of FITC-labeled GluA2 peptide with increasing concentrations of unlabeled GluA2 and compound 1o using recombinant full-length PICK1.

C, Fluorescence polarization binding of FITC-labeled GluN2B peptide at increasing concentrations of unlabeled GluN2B and compound 1o using purified PSD95 PDZ 1-2 proteins.

D, Fluorescence polarization binding of FITC-labeled GKAP peptide at increasing concentrations of unlabeled GKAP and compound 1o using purified Shank3 PDZ protein.

E, Sequence alignment of PDZ domains of the target and off-target with specific residues discussed in the S_0-S_{-1}-S_{-2} pocket highlighted in black boxes (Phe53, Lys83, Ala87, and Gln91). Residues that are identical are highlighted with dark blue columns. ***P < 0.001. FITC, fluorescein isothiocyanate; GluN2B, N-methyl-D-aspartate receptor subunit 2B; PSD95, postsynaptic density-95; GKAP, guanylate kinase-associated protein.

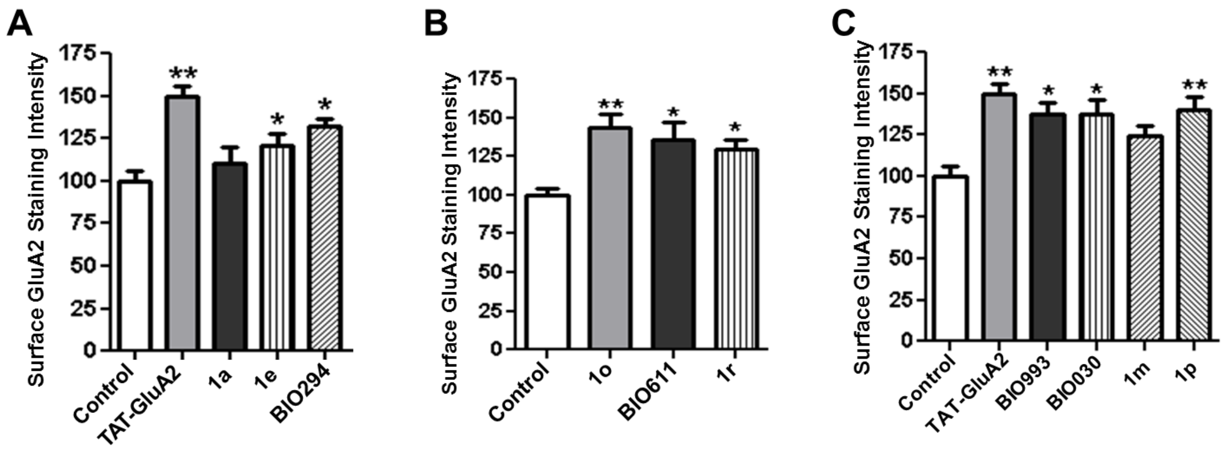

Supplemental Figure 4. PICK1 inhibitors stabilize surface GluA2 in cultured neurons.

A-C, Histograms showing quantification of staining intensities of surface GluA2 in cultured neurons treated with a series of PICK1 compounds normalized to control (untreated) n = n = 14 (*P < 0.05). See Supplemental Figure 7 for compound structures not in Figure 2.

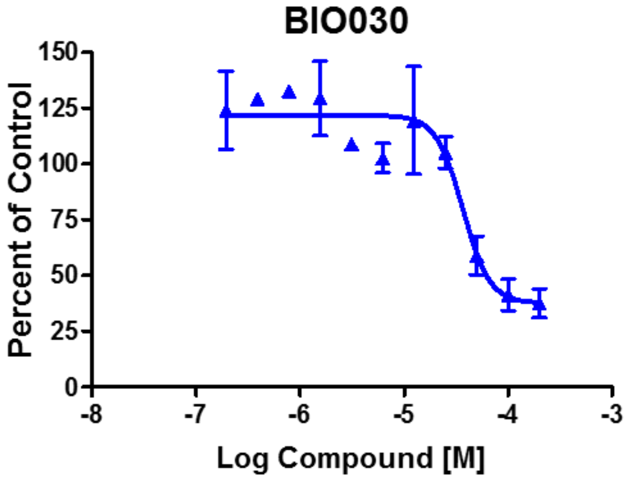

Supplemental Figure 5. PICK1 compounds block Aβ42-mediated increase in intracellular calcium levels in a concentration-dependent manner.

PICK1 inhibitor BIO030 blocks Aβ42-induced elevation in intracellular calcium concentrations in cultured neurons. See Supplemental Figure 7 for compound structure.

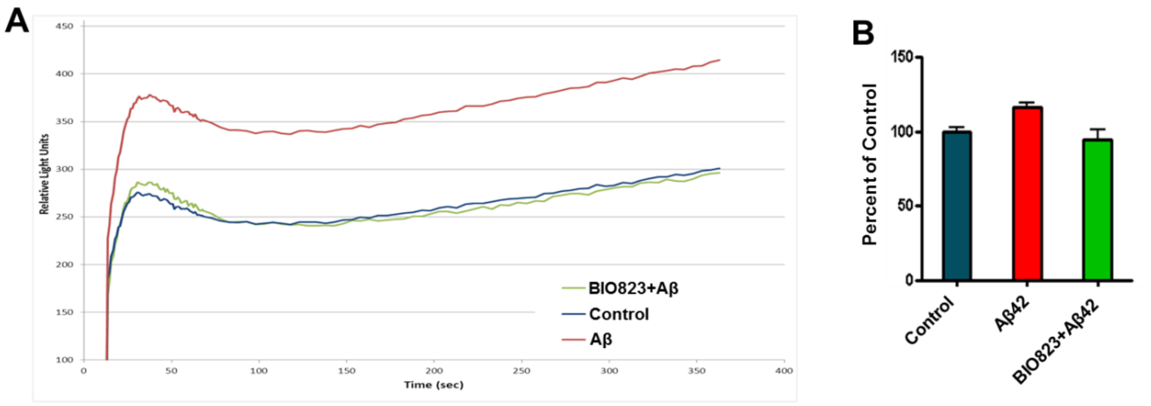

Supplemental Figure 6. PICK1 inhibitor blocks Aβ42-induced increase in intracellular calcium concentration in neurons.

A, AMPA receptor-dependent calcium influx was measured in control neurons (untreated) or neurons treated with Aβ42 in the absence (Aβ42) or presence of the PICK1 inhibitor BIO823 (Aβ42 + BIO823).

B, Histograms showing quantification of intracellular calcium levels comparing Aβ42-treated with Aβ42+BIO823-treated neurons normalized to control values.

See Supplemental Figure 7 for compound structure.

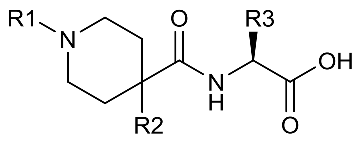

Compound	R1	R2	R3	IC50 (uM)
BIO294	N-AcPro	Ph	iPr	26.2 (1)
BIO030	3-CF_3PhCH_2	4-BrPh	iPr	1.661 (1)
BIO611	2-$FPhCH_2$	4-BrPh	CH_2cPr	2.17
BIO823	2,6-Cl_2PhCH_2	4-BrPh	CH_2cPr	0.216 (1)
BIO993	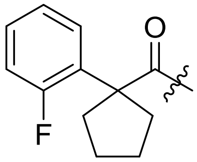	4-BrPh	iPr	2.426 (1)

Supplemental Figure 7. Structures of Additional PICK1 Small Molecule Inhibitors. Structures and biochemical potency of additional PICK1 small molecule inhibitors.

(1) IC50 determined using earlier generation tracer BIO426 in place of BIO424.

BIO426 is 5-[[6-[(2S)-2-[4-(4-bromophenyl)-4-[[(1S)-1-carboxy-2-cyclopropyl-ethyl]carbamoyl]piperidine-1-carbonyl]pyrrolidin-1-yl]-6-oxo-hexyl]carbamothioylamino]-2-(3-hydroxy-6-oxo-xanthen-9-yl)benzoic acid

Supplemental Table 1. Small molecule screening data

Category	Parameter	Description
Assay	*In vitro*	Fluorescence polarization
	Target	PICK1 (His-MBP-1-356)
	Detection of FITC-GluR2**(V*YGIESVKI)***	
	PICK1 1-356 (produced in baculovirus)	
	See assay methods	
Library	260,000 lead like, 13,000 fragments	
	Evotec	
Screen	2080-well plates	
	50μM lead-like (1% DMSO);	
	250μM fragments (6.25%DMSO)	
	Unlabelled GluR2 peptide control	
	Reagent/compound dispensing system	
	Envision 2103 Multilabel Readers, Perkin Elmer	
	Z'=0.75	
	Inhjbition activities were in range of 20-140% compared to control (unlabeled GluR2 Peptide)	
Post-HTS analysis	Repetition and confirmation at 50 μM	
	189 confirmed out of 934 at hit rate of 20%	
	No counter-screens.	
	Repurchased hits to confirm purity	

Supplemental Table 2. Data collection and refinement statistics (molecular replacement)

Data Collection	PICK1-PDZ-596
Space Group	$P3_2$
Cell Dimensions	
a (Å)	54.31
b (Å)	54.31
c (Å)	78.63
Wavelength (Å)	0.98
Resolution (Å)	50-1.69
R_{sym} [a]	0.06 (1.2)
I/σ	7.1 (1.5)
Multiplicity	5.1
Total No. reflections/ No. unique reflections	28,521/2,831
Mean I/σ	16.4 (1.1)
Completeness (%)	99.3 (99.4)
Rwork (Rfree)	15.54/17.79
$CC_{1/2}$	0.99 (0.49)
No. Molecules per asymmetric unit	2
R.m.s.d. bond distance (Å)	0.032
R.m.s.d bond angle (deg)	2.73
Total no. of non-H atoms in ASU	1,5945
No. of solvent molecules	247
Avg. protein B-value ($Å^2$)	27.64
Avg. solvent B-value ($Å^2$)	37.41
Ramachandran Plot<br>Preferred<br>Generous<br>Disallowed	 99.41<br>0.59<br>0.0

1 crystal used for this structure. Values in parentheses are for highest-resolution shell.

Synthesis: Standard amide coupling conditions

Into a solution of acid (1.0 mmol) and amine (1.0 mmol, 1.0 eq) in dichloromethane (6 mL) were added 1-hydroxybenzotriazole (203 mg, 1.5 mmol, 1.5 eq), triethylamine (303 mg, 3.0 mmol, 3.0 eq), and 1-ethyl-3-(3-dimethylaminopropyl)carbodiimide hydrochloride (239 mg, 1.3 mmol, 1.3 eq). The reaction mixture was stirred at room temperature for 10 h and diluted with dichloromethane (40 mL). The organic layer was washed with water (40 mL) and concentrated. The residue was purified by column chromatography on silica gel (using petroleum ether/ethyl acetate as eluent) to afford the coupled product.

Synthesis: Standard ester hydrolysis conditions

Into a solution of ester (0.48 mmol) in 5 mL of tetrahydrofuran/water (1:1) was added lithium hydroxide monohydrate (40 mg, 0.96 mmol, 2.0 eq). The mixture was stirred at room temperature for 2 h and diluted with 15 mL of water. The aqueous layer was washed with diethyl ether (15 mL×2) and acidified with acetic acid to pH 5. The resulting mixture was extracted with dichloromethane (20 mL×3). The combined organic phases were dried and concentrated to give the acid.

Synthesis of (2S)-2-[[1-[(2S)-1-tert-butoxycarbonylpyrrolidine-2-carbonyl]-4-phenyl-piperidine-4-carbonyl]amino]-3-methyl-butanoic acid [1a]

Into a suspension of 4-phenylpiperidine-4-carboxylic acid (**2a**) tosylate salt (10 g, 26.5 mmol) and triethylamine (11.1 mL, 79.5 mmol, 3.0 eq) in tetrahydrofuran (120 mL) was added di-tert-butyl dicarbonate (6.36 g, 29.1 mmol, 1.1 eq). The reaction was heated at reflux for 1 h and then cooled. The solvent was removed and the residue was dissolved in 200 mL of water and basified with 10 M sodium hydroxide to pH 9-10. The aqueous layer was washed with diethyl ether (100 mL×2) and acidified with acetic acid to pH 5. The white solid was collected by filtration to give **3a** as a white solid (1.8 g, Y: 98%).

ESI-MS (M+H)+: 327.9.

1H NMR (400 MHz, DMSO-d6) δ: 12.45 (br s, 1H), 7.40-7.25 (m, 5H), 3.82-3.78 (m, 2H), 2.96-2.94 (m, 2H), 2.38-2.34 (m, 2H), 1.71-1.64 (m, 2H), 1.39 (s, 9H).

3a (305 mg, 1.0 mmol) and L-valine methyl ester (168 mg, 1.0 mmol, 1.0 eq) were reacted using the standard amide coupling conditions to afford **4a** as a white solid (350 mg, Y: 80%).

ESI-MS (M+H)+: 441.3.

1H NMR (400 MHz, CDCl3) δ: 7.40-7.29 (m, 5H), 5.62-5.60 (m, 1H), 4.47-4.44 (m, 1H), 3.66 (s, 3H), 3.62-3.40 (m, 4H), 2.43-2.32 (m, 2H), 2.13-1.98 (m, 3H), 1.44 (s, 9H), 0.75 (d, J = 6.8 Hz, 3H), 0.64 (d, J = 6.8 Hz, 3H).

The mixture of **4a** (1.70 g, 4 mmol) in 10 mL of trifluoroacetic acid/dichloromethane (1:1) was stirred at room temperature for 7 h and concentrated to give **5a** as trifluoroacetate salt which was directly used for next step without further purification (1.72 g, Y: 100%).

ESI-MS (M+H)+: 319.2.

1H NMR (400 MHz, CDCl3) δ: 7.40-7.37 (m, 4H), 7.31-7.26 (m, 1H), 5.67-5.65 (m, 1H), 4.46-4.44 (m, 1H), 3.67 (s, 3H), 3.07-2.98 (m, 3H), 2.76-2.74 (m, 2H), 2.45-2.37 (m, 2H), 2.20-2.17 (m, 1H), 2.08-2.03 (m, 2H), 0.74 (d, J = 7.2 Hz, 3H), 0.65 (d, J = 6.8 Hz, 3H).

5a was converted to **1a** methyl ester using the standard amide coupling conditions (1.7 g, Y: 90%).

ESI-MS (M+H)+: 516.3.

1H NMR (400 MHz, CDCl3) δ: 7.39-7.30 (m, 5H), 5.61-5.59 (m, 1H), 4.69-4.55 (m, 1H), 4.47-4.44 (m, 1H), 4.20-3.85 (m, 1H), 3.69-3.40 (m, 8H), 2.60-2.32 (m, 2H), 2.23-1.91 (m, 7H), 1.44-1.28 (m, 9H), 0.78-0.58 (m, 6H).

1a methyl ester was converted to **1a** using standard ester hydrolysis conditions (210 mg, Y: 84%).

ESI-MS (M+H)+: 501.9.

1H NMR (400 MHz, DMSO-d6) δ: 12.43 (br, 1H), 7.42-4.23 (m, 6H), 4.65-4.60 (m, 1H), 4.08-3.65 (m, 3H), 3.32-3.26 (m, 4H), 3.08-2.60 (m, 2H), 2.02-1.90 (m, 2H), 1.79-1.67 (m, 5H), 1.37-1.24 (m, 9H), 0.78-0.58 (m, 6H).

Synthesis of **1b** - **1n** by analogy to **1a**. For non-commercially available core, the piperidines **2** were synthesized by reaction of tert-butyl bis(2-chloroethyl)carbamate with the appropriate substituted acetonitrile, followed by acidic hydrolysis.

(2S)-2-[[1-[(2S)-1-tert-butoxycarbonylpyrrolidine-2-carbonyl]-4-cyclohexyl-piperidine-4-carbonyl]amino]-3-methyl-butanoic acid [1b]

ESI-MS (M+H)+: 508.3

1H NMR (400 MHz, CDCl3) δ: 4.74-4.67 (m, 1H), 4.44-4.41 (m, 1H), 4.35-4.32 (m, 1H), 3.96-3.93 (m, 1H), 3.53-3.42 (m, 2H), 3.25-3.18 (m, 1H), 2.82-2.67 (m, 1H), 2.31-2.17 (m, 4H), 1.92-1.67 (m, 7H), 1.54-1.46 (m, 1H), 1.48-1.37 (m, 10H), 1.31-1.15 (m, 4H), 1.13-1.06 (m, 2H), 1.03-0.99 (m, 6H), 0.93-0.90 (m, 1H).

(2S)-2-[[1-[(2S)-1-tert-butoxycarbonylpyrrolidine-2-carbonyl]-4-morpholino-piperidine-4-carbonyl]amino]-3-methyl-butanoic acid [1c]

ESI-MS (M+1)+: 511.3

1H NMR (400 MHz, CD3OD) δ: 4.63-4.57 (m, 1H), 4.22-4.11 (m, 2H), 3.83-3.78 (m, 1H), 3.66-3.60 (m, 4H), 3.42-3.31 (m, 3H), 2.96-2.93 (m, 1H), 2.52-2.42 (m, 4H), 2.20-2.10 (m, 2H), 1.98-1.68 (m, 7H), 1.37-1.19 (m, 9H), 0.90-0.83 (m, 6H).

(2S)-2-[[4-benzyl-1-[(2S)-1-tert-butoxycarbonylpyrrolidine-2-carbonyl]piperidine-4-carbonyl]amino]-3-methyl-butanoic acid [1d]

ESI-MS (M+H) +: 516.3.

1H NMR (400 MHz, CD3OD) δ: 7.22-7.16 (m, 3H), 7.15-7.11 (m, 2H), 4.72-4.64 (m, 1H), 4.36-4.22 (m, 2H), 3.92-3.88 (m, 1H), 3.52-3.40 (m, 2H), 3.28-3.20 (m, 1H), 3.05-2.78 (m, 3H), 2.28-2.11 (m, 4H), 1.95-1.76 (m, 3H), 1.55-1.35 (m, 11H), 0.97-0.92 (m, 6H).

(2S)-2-[[4-(4-bromophenyl)-1-[(2S)-1-tert-butoxycarbonylpyrrolidine-2-carbonyl]piperidine-4-carbonyl]amino]-3-methyl-butanoic acid [1e]

ESI-MS (M+H)+: 580.2

1H NMR (400 MHz, DMSO-d6) δ: 7.53-7.50 (m, 2H), 7.43-7.31 (m, 3H), 4.69-4.60 (m, 1H), 4.20-3.96 (m, 2H), 3.87-3.72 (m, 1H), 3.33-3.27 (m, 2H), 3.19-2.82 (m, 1H), 2.66-2.58 (m, 1H), 2.45-2.43 (m, 1H), 2.21-2.01 (m, 2H), 1.98-1.60 (m, 5H), 1.37-1.23 (m, 10H), 0.77-0.70 (m, 6H).

(2S)-2-[[1-[(2S)-1-tert-butoxycarbonylpyrrolidine-2-carbonyl]-4-[4-(trifluoromethyl)phenyl]piperidine-4-carbonyl]amino]-3-methyl-butanoic acid [1f]

ESI-MS (M+H)+: 570.2

1H NMR (400 MHz, DMSO-d6) δ: 7.71-7.69 (m, 2H), 7.64-7.55 (m, 3H), 4.70-4.60 (m, 1H), 4.25-4.03 (m, 2H), 3.91-3.78 (m, 1H), 3.06-3.00 (m, 1H), 2.79-2.59 (m, 2H), 2.22-1.98 (m, 2H), 1.91-1.58 (m, 6H), 1.38-1.20 (m, 11H), 0.79-0.70 (m, 6H).

2-[[4-(4-bromophenyl)-1-[(2S)-1-tert-butoxycarbonylpyrrolidine-2-carbonyl]piperidine-4-carbonyl]amino]acetic acid [1g]

ESI-MS (M+H)+: 538.1

(2S)-2-[[4-(4-bromophenyl)-1-[(2S)-1-tert-butoxycarbonylpyrrolidine-2-carbonyl]piperidine-4-carbonyl]amino]propanoic acid [1h]

ESI-MS (M+H)+: 552.2

2-[[4-(4-bromophenyl)-1-[(2S)-1-tert-butoxycarbonylpyrrolidine-2-carbonyl]piperidine-4-carbonyl]amino]-2-methyl-propanoic acid [1i]

ESI-MS (M+H)+: 568.1 (for Br-81)

(2S)-2-[[4-(4-bromophenyl)-1-[(2S)-1-tert-butoxycarbonylpyrrolidine-2-carbonyl]piperidine-4-carbonyl]amino]-3-(1H-indol-3-yl)propanoic acid [1j]

ESI-MS (M+H)+: 567.2 (for des-Boc)

(2S)-2-[[4-(4-bromophenyl)-1-[(2S)-1-tert-butoxycarbonylpyrrolidine-2-carbonyl]piperidine-4-carbonyl]amino]-3-(1H-imidazol-4-yl)propanoic acid [1k]

ESI-MS (M+H)+: 618.2

(2S)-2-[[4-(4-bromophenyl)-1-[(2S)-1-tert-butoxycarbonylpyrrolidine-2-carbonyl]piperidine-4-carbonyl]amino]-2-cyclopentyl-acetic acid [1l]

ESI-MS (M+H)+: 605.7

(2S)-2-[[4-(4-bromophenyl)-1-[(2S)-1-tert-butoxycarbonylpyrrolidine-2-carbonyl]piperidine-4-carbonyl]amino]-3-cyclopropyl-propanoic acid [1m]

ESI-MS (M+Na)+: 614.2 (for M+Na)

(2S)-2-[[1-[(2S)-1-tert-butoxycarbonylpyrrolidine-2-carbonyl]-4-[4-(trifluoromethyl)phenyl]piperidine-4-carbonyl]amino]-3-cyclopropyl-propanoic acid [1n]

ESI-MS (M+H)+: 582.3.

1H NMR (400 MHz, CD3OD) δ: 7.47-7.44 (m, 4H), 4.54-4.45 (m, 1H), 4.19-4.13 (m, 1H), 4.02-3.97 (m, 1H), 3.75-3.60 (m, 1H), 3.38-3.17(m, 3H), 2.99-2.93 (m, 1H), 2.52-2.40 (m, 2H), 2.07-2.06 (m, 1H), 1.92-1.55 (m, 5H), 1.41-1.32 (m, 2H), 1.22-1.11 (m, 9H), 0.27-0.23 (m, 1H), 0.07-0.01 (m, 2H),-0.24--0.37 (m, 2H).

Synthesis of (2S)-2-[[4-(4-bromophenyl)-1-[[2-(trifluoromethyl)phenyl]methyl]piperidine-4-carbonyl]amino]-3-cyclopropyl-propanoic acid [1o]

Methyl (S)-2-(4-(4-bromophenyl)piperidine-4-carboxamido)-3-cyclopropylpropanoate **5m** (102 mg, 0.25 mmol) and 2-(trifluoromethyl)benzaldehyde (66 mg, 0.38 mmol, 1.5 eq) were dissolved in 1,2-dichloroethane (8 mL). The mixture was refluxed for 2 h. After cooling to room temperature, sodium cyanoborohydride (32 mg, 0.50 mmol, 2.0 eq) was added and stirred at room temperature for 2 h. Water (5 mL) was added to quench the reaction. The organic phase was washed with brine and dried over sodium sulfate. After

concentration, the residue was dissolved in tetrahydrofuran/water (4 mL, 3/1). Lithium hydroxide monohydrate (32 mg, 0.75 mmol, 3.0 eq) was added and stirred at 0 ^{o}C for 16 h. The mixture was adjusted pH 5-6 with 1N hydrochloric acid and extracted with dichloromethane (15 mL × 2). The combined organic phase was washed with brine and dried over Na_2SO_4. After concentration, the residue was purified by preparative HPLC (0.5% TFA/H_2O) to give **1o** as a white solid (37 mg, Y: 27%).

ESI-MS (M+H)+: 555.1 (for Br-81)

1H NMR (400 MHz, CD3OD) δ: 7.92-7.89 (m, 2H), 7.82 (t, J = 7.6 Hz, 1H), 7.73 (t, J = 7.6 Hz, 1H), 7.56 (d, J = 8.4 Hz, 2H), 7.37 (d, J = 8.4 Hz, 2H), 4.55-4.46 (m, 3H), 3.60-3.49 (m, 4H), 2.99-2.79 (m, 2H), 2.46-2.42 (m, 1H), 2.22-2.18 (m, 1H), 1.73-1.64 (m, 2H), 0.56-0.52 (m, 1H), 0.35-0.31 (m, 2H), 0.01-0.00 (m, 2H).

Analogs **1p** - **1q** were synthesized as per **1o**.

(2S)-2-[[4-(4-bromophenyl)-1-[[3-(trifluoromethyl)phenyl]methyl]piperidine-4-carbonyl]amino]-3-cyclopropyl-propanoic acid [1p]

ESI-MS (M+H)+: 553.0

1H NMR (400 MHz, CD3OD) δ: 7.80 (s, 1H), 7.71 (d, 2H), 7.42-7.40 (m, 1H), 7.28 (d, J= 8.4 Hz, 2H), 7.13 (d, J= 8.4 Hz, 2H), 4.13-4.10 (m, 1H), 3.84 (s, 2H), 3.04-2.73 (m, 4H), 2.51-2.44 (m, 2H), 2.03-2.02 (m,1H),1.80-1.76 (m,1H),1.42-1.32 (m, 2H), 0.23-0.18 (m,1H), 0.01 (d, J= 8.4 Hz, 2H), -0.07--0.14 (m, 2H).

(2S)-2-[[4-(4-bromophenyl)-1-[[4-(trifluoromethyl)phenyl]methyl]piperidine-4-carbonyl]amino]-3-cyclopropyl-propanoic acid; 2,2,2-trifluoroacetic acid [1q]

ESI-MS (M+H)+: 553.0

1H NMR (400 MHz, CD3OD) δ: 7.86 (d, J = 8.0 Hz, 2H), 7.77 (d, J = 8.0 Hz, 2H), 7.60 (d, J = 8.8 Hz, 2H), 7.41 (d, J = 8.4 Hz, 2H), 4.49-4.46 (m, 1H), 4.42 (s, 2H), 3.49-3.39 (m, 4H), 2.91-2.79 (m, 2H), 2.39-2.36 (m, 1H), 2.11-2.08 (m, 1H), 1.71-1.63 (m, 2H), 0.54-0.51 (m, 1H), 0.35-0.32 (m , 2H), 0.01-0.00 (m, 2H).

Synthesis of (2S)-2-[[4-(4-bromophenyl)-1-[1-[2-(trifluoromethyl)phenyl]ethyl]piperidine-4-carbonyl]amino]-3-cyclopropyl-propanoic acid [1r]

A mixture of methyl (S)-2-(4-(4-bromophenyl)piperidine-4-carboxamido)-3-cyclopropylpropanoate **5m** (408 mg, 1.0 mmol, 1.0 eq), 2'-(trifluoromethyl)acetophenone (282 mg, 1.5 mmol, 1.5 eq), and titanium(IV) ethoxide (570 mg, 2.5 mmol, 2.5 eq) in THF (5 mL) was stirred at 100 ^oC (microwave) for 2 h. After cooling to room temperature, sodium cyanoborohydride (126 mg, 2.0 mmol, 2.0 eq) was added to the mixture. The mixture was stirred at room temperaturert for 16 h. The mixture was diluted with water (20 mL) and extracted with ethyl acetate (2 × 30 mL). The combined organic phase was washed with brine and dried over sodium sulfate. After filtration and concentration, the residue was dissolved in tetrahydrofuran/water (10 mL/2 mL). Lithium hydroxide monohydrate (84 mg, 2.0 mmol, 2.0 eq) was added to the mixture. The mixture was stirred at room temperature for 1 h. The mixture was adjusted to pH = 5-6 and extracted with ethyl acetate (2 × 30 mL). The combined organic phase was washed with brine and dried over sodium sulfate. After filtration and concentration, the residue was purified by preparative-HPLC (MeOH/H_2O with 0.05% NH_4HCO_3 as mobile phase from 5% to 95%) to give **1r** (53 mg, Y: 11%).

ESI-MS (M+H)+: 567.1

1H NMR (400 MHz, CD3OD) δ: 7.60 (d, J = 7.6 Hz, 1H), 7.76-7.70 (m, 2H), 7.55-7.52 (m, 3H), 7.39 (d, J = 8.4 Hz, 2H), 4.42-4.41 (m, 1H), 4.00-3.98 (m, 1H), 3.32-3.30 (m, 1H), 2.75-2.57 (m, 5H), 1.70-1.49 (m, 5H), 0.53-0.51 (m, 1H), 0.32-0.30 (m, 2H), 0.00- -0.04 (m, 2H).

(2S)-3-cyclopropyl-2-[[1-phenyl-4-[4-(trifluoromethyl)phenyl]piperidine-4-carbonyl]amino]propanoic acid [1s]

A mixture of methyl (S)-3-cyclopropyl-2-(4-(4-(trifluoromethyl)phenyl)piperidine-4-carboxamido)propanoate **5s** (100 mg, 0.25 mmol, 1.0 eq), bromobenzene (47 mg, 0.30 mmol, 1.20 eq), sodium hydroxide (240 mg, 2.5 mmol, 2.0 eq), tris(dibenzylideneacetone)dipalladium(0) (46 mg, 0.01 mmol, 0.05 eq), and 2-

dicyclohexylphosphino-2′,6′-dimethoxybiphenyl SPhos (9 mg, 0.02 mmol, 0.10 eq) was added to toluene (2 mL). The mixture was stirred at 100 ºC for 16 h. Then water (50 mL) was added and extracted with ethyl acetate (80 mL × 2). The combined organics were washed with brine (50 mL), concentrated, and purified by preparative HPLC (0.5% TFA) to give **1s** as a white solid (16 mg, Y: 16%).

ESI-MS (M+H)+: 461.1.

1H NMR (400 MHz, CDCl3) δ: 7.52-7.50 (m, 4H), 7.16 (t, J = 8.0 Hz, 2H), 6.82-6.76 (m, 3H), 6.43-6.41 (m, 1H), 3.99-3.97 (m, 1H), 3.39-3.37 (m, 1H), 3.25-3.23 (m, 2H), 2.99-2.97 (m, 1H), 2.46-2.43 (m, 2H), 2.35-2.29 (m, 2H), 1.45-1.43 (m, 2H), 0.23-0.21 (m, 1H), 0.04-0.02 (m, 2H), -0.36--0.38 (m, 2H).

Synthesis of (2S)-3-cyclopropyl-2-[[1-(1-naphthyl)-4-[4-(trifluoromethyl)phenyl]piperidine-4-carbonyl]amino]propanoic acid [1t]

A mixture of methyl (S)-3-cyclopropyl-2-(4-(4-(trifluoromethyl)phenyl)piperidine-4-carboxamido)propanoate **5s** (0.1002 g, 0.2515 mmol), 1-bromonaphthalene (0.07811 g, 0.3772 mmol), palladium acetate (0.00565 g, 0.0251 mmol), cesium carbonate (0.246 g, 0.754 mmol), and (R)-(+)-2,2'-bis(diphenylphosphino)-1,1'-binaphthyl (0.0156 g, 0.0251 mmol) was added to toluene (2.5 mL). The reaction flask was purged with nitrogen, and the mixture was allowed to stir for 16 hrs at 100 °C. The reaction mixture was taken up in ethyl acetate and washed with water. The organic phases were combined, washed with saturated sodium chloride, dried over sodium sulfate, and concentrated. The residue was then taken up in methylene chloride and purified by silica gel chromatography using ethyl acetate in hexanes as eluent to give **1t** methyl ester (60.7 mg, Y: 46%)

ESI-MS (M+H)+: 525.2

1t methyl ester (60.7 mg, 0.12 mmol) was then taken up in tetrahydrofuran (1 mL) and methanol (1 mL), and 2.0 M of lithium hydroxide in water (1 mL, 2 mmol) was then added. The reaction mixture was allowed to stir for 1 hr at room temperature, and then

the solvent was evaporated. The residue was then taken up in DMSO, neutralized with TFA, and purified by preparative HPLC, giving the product, **1t** (36.3 mg, Y: 28%).

ESI-MS (M+H)+: 511.0

1H NMR (DMSO-d6) δ: 8.16 (d, J = 7.3 Hz, 1H), 7.84 - 7.97 (m, 2H), 7.67 - 7.79 (m, 4H), 7.57 (d, J = 8.0 Hz, 1H), 7.44 - 7.54 (m, 2H), 7.40 (t, J = 7.8 Hz, 1H), 7.05 (d, J = 7.3 Hz, 1H), 4.27 - 4.38 (m, 1H), 3.57 (br. s., 1H), 2.91 - 3.14 (m, 2H), 2.72 - 2.88 (m, 2H), 2.15 - 2.34 (m, 2H), 1.42 - 1.72 (m, 2H), 0.46 - 0.59 (m, 1H), 0.16 - 0.28 (m, 2H), -0.11 - 0.05 (m, 3H)

N-Aryl analogs **1u** – **1v** synthesized as per **1t.**

(2S)-3-cyclopropyl-2-[[1-(8-quinolyl)-4-[4-(trifluoromethyl)phenyl]piperidine-4-carbonyl]amino]propanoic acid [1u]

ESI-MS (M+H)+: 512.1

1H NMR (400 MHz, DMSO-d6) δ 8.87 (dd, J=1.76, 4.02 Hz, 1H), 8.28 (dd, J=1.51, 8.28 Hz, 1H), 7.75 (br s, 1H), 7.71 (s, 4H), 7.46-7.52 (m, 2H), 7.41-7.46 (m, 1H), 7.10 (br d, J=6.02 Hz, 1H), 3.75 (br t, J=11.42 Hz, 2H), 3.05-3.21 (m, 2H), 2.64-2.78 (m, 2H), 2.14-2.27 (m, 2H), 1.57-1.67 (m, 1H), 1.41-1.51 (m, 1H), 0.44-0.54 (m, 1H), 0.11-0.23 (m, 2H), -0.08-0.01 (m, 1H), -0.19--0.09 (m, 1H)

(2S)-3-cyclopropyl-2-[[1-(6-methyl-8-quinolyl)-4-[4-(trifluoromethyl)phenyl]piperidine-4-carbonyl]amino]propanoic acid [1v]

ESI-MS (M+H)+: 526.0

1H NMR (DMSO-d6) δ: 8.94 (d, J = 2.8 Hz, 1H), 8.51 (br. s., 1H), 7.95 (d, J = 6.3 Hz, 1H), 7.64 - 7.80 (m, 6H), 7.59 (br. s., 1H), 7.36 (br. s., 1H), 4.26 - 4.41 (m, 1H), 3.14 - 3.43 (m, 2H), 2.70 - 2.90 (m, 2H), 2.24 - 2.45 (m, 2H), 1.43 - 1.72 (m, 2H), 0.42 - 0.63 (m, 1H), 0.21 (ddd, J = 12.7, 8.2, 4.4 Hz, 3H), -0.12 - 0.06 (m, 3H)

(2S)-3-cyclopropyl-2-[[1-(6-ethyl-8-quinolyl)-4-[4-(trifluoromethyl)phenyl]piperidine-4-carbonyl]amino]propanoic acid [1w]

ESI-MS (M-H)-: 538.3

(2S)-3-cyclopropyl-2-[[1-(6-isopropyl-8-quinolyl)-4-[4-(trifluoromethyl)phenyl]piperidine-4-carbonyl]amino]propanoic acid [1x]

ESI-MS (M-H)-: 552.3

(2S)-3-cyclopropyl-2-[[1-(6-fluoro-8-quinolyl)-4-[4-(trifluoromethyl)phenyl]piperidine-4-carbonyl]amino]propanoic acid [1y]

ESI-MS (M+H)+: 530.0

1H NMR (DMSO-d6) δ: 8.81 - 8.88 (m, 1H), 8.32 (d, J = 7.5 Hz, 1H), 7.90 (d, J = 7.8 Hz, 1H), 7.66 - 7.77 (m, 4H), 7.57 (dd, J = 8.3, 4.3 Hz, 1H), 7.29 (dd, J = 8.7, 2.1 Hz, 1H), 6.99 (d, J = 9.5 Hz, 1H), 4.22 - 4.37 (m, 1H), 3.87 (t, J = 12.9 Hz, 2H), 3.05 - 3.29 (m, 2H), 2.76 (t, J = 13.6 Hz, 2H), 2.10 - 2.31 (m, 2H), 1.41 - 1.72 (m, 2H), 0.46 - 0.56 (m, 1H), 0.14 - 0.28 (m, 2H), -0.13 - 0.07 (m, 2H)

(2S)-2-[[1-(6-chloro-8-quinolyl)-4-[4-(trifluoromethyl)phenyl]piperidine-4-carbonyl]amino]-3-cyclopropyl-propanoic acid [1z, BIO922]

ESI-MS (M+H)+: 546.1

1H NMR (400 MHz, DMSO-d6) δ 12.44 (br s, 1H), 8.87 (dd, J=1.76, 4.27 Hz, 1H), 8.26 (dd, J=1.63, 8.41 Hz, 1H), 7.88 (br d, J=7.78 Hz, 1H), 7.71 (s, 4H), 7.59 (d, J=2.01 Hz, 1H), 7.49-7.57 (m, 1H), 6.99 (d, J=2.01 Hz, 1H), 4.24-4.33 (m, 1H), 3.85 (br s, 2H), 3.20 (br t, J=10.92 Hz, 1H), 3.09 (br t, J=10.67 Hz, 1H), 2.75 (br t, J=13.80 Hz, 2H), 2.10-2.25 (m, 2H), 1.59-1.70 (m, 1H), 1.42-1.53 (m, 1H), 0.50 (br d, J=6.53 Hz, 1H), 0.14-0.26 (m, 2H), -0.04-0.04 (m, 1H), -0.12--0.04 (m, 1H)

(2S)-2-[[1-(6-bromo-8-quinolyl)-4-[4-(trifluoromethyl)phenyl]piperidine-4-carbonyl]amino]-3-cyclopropyl-propanoic acid [1aa]

ESI-MS (M-H)-: 588.1

(2S)-3-cyclopropyl-2-[[4-[4-(trifluoromethyl)phenyl]-1-[6-(trifluoromethyl)-8-quinolyl]piperidine-4-carbonyl]amino]propanoic acid [1ab]

ESI-MS (M-H)-: 578.2

(2S)-3-cyclopropyl-2-[[1-(6-methoxy-8-quinolyl)-4-[4-(trifluoromethyl)phenyl]piperidine-4-carbonyl]amino]propanoic acid [1ac]

ESI-MS (M-H)-: 540.2

(2S)-3-cyclopropyl-2-[[1-[6-(trifluoromethoxy)-8-quinolyl]-4-[4-(trifluoromethyl)phenyl]piperidine-4-carbonyl]amino]propanoic acid [1ad]

ESI-MS (M+H)+: 596.0

1H NMR (DMSO-d6) δ: 8.91 (dd, J = 4.0, 1.8 Hz, 1H), 8.40 (dd, J = 8.3, 1.5 Hz, 1H), 7.91 (d, J = 7.5 Hz, 1H), 7.71 (s, 4H), 7.60 (dd, J = 8.3, 4.3 Hz, 1H), 7.50 (br. s., 1H), 6.95 (s, 1H), 4.31 (d, J = 2.5 Hz, 1H), 3.89 (br. s., 2H), 3.05 - 3.29 (m, 2H), 2.67 - 2.85 (m, 2H), 2.19 (d, J = 13.8 Hz, 2H), 1.39 - 1.74 (m, 2H), 0.46 - 0.57 (m, 1H), 0.21 (d, J = 7.5 Hz, 2H), -0.11 - 0.05 (m, 2H)

5-[[6-[(2S)-2-[4-[[(1S)-1-carboxy-2-cyclopropyl-ethyl]carbamoyl]-4-[4-(trifluoromethyl)phenyl]piperidine-1-carbonyl]pyrrolidin-1-yl]-6-oxo-hexyl]carbamothioylamino]-2-(3-hydroxy-6-oxo-xanthen-9-yl)benzoic acid [BIO424 tracer]

BIO424 was synthesized in 5 steps from **1n** methyl ester, Boc-ε-aminocaproic acid [CAS 6404-29-1], fluorescein isothiocyanate (FITC) [CAS 27072-45-3].

ESI-MS (M+H)+: 985.3

1H NMR (400 MHz, METHANOL-d4) δ 8.06 - 8.13 (m, 1H), 7.63 - 7.81 (m, 1H), 7.59 (s, 4H), 7.08 - 7.14 (m, 1H), 6.66 (br. s., 4H), 6.53 (br. s., 2H), 4.37 - 4.45 (m, 1H), 4.15 - 4.30 (m, 1H), 3.82 - 4.03 (m, 1H), 3.61 (br. s., 5H), 2.97 - 3.22 (m, 1H), 2.47 - 2.76 (m, 2H), 2.28 - 2.45 (m, 2H), 2.11 - 2.26 (m, 1H), 1.88 - 2.07 (m, 2H), 1.72 - 1.88 (m, 2H), 1.64 (br. s., 6H), 1.35 - 1.47 (m, 2H), 1.20 - 1.35 (m, 2H), 0.33 - 0.51 (m, 1H), 0.12 - 0.30 (m, 2H), -0.17 - -0.03 (m, 2H)

Recombinant proteins:

PICK1 PDZ-QSAV was constructed as follows: cDNA for human PICK1 encoding residues 1 to 105 was cloned into NcoI-XhoI-digested pET15b with a TEV cleavable N-terminal HIS tag and the C-terminal fused NKLQQSAV tail (PICK1 PDZ-QSAV). BL21 (DE3) E. coli cells transformed with PICK1 PDZ-QSAV were grown at 37°C in LB media supplemented with ampicillin to an OD of 1, at which point the temperature was reduced to 18°C and protein expression was induced with 1 mM IPTG. After 16 hours, the cells were harvested and resuspended in lysis buffer (25 mM Tris-HCl pH 8.0, 250 mM NaCl, 10% (v/v) glycerol, 2.5 mM β-mercaptoethanol, 20 mM imidazole, and Roche EDTA-free protease inhibitor cocktail) and subjected to two passes through a microfluidizer (Microfluidics, Newton, MA) at 4°C. The lysate was clarified by centrifugation at 20,000 x g for 1 hour at °C, and PICK1 PDZ-QSAV was captured by batch binding to nickel resin overnight at 4°C. The nickel resin was washed with buffer A (25 mM Tris-HCl pH 8.0, 250 mM NaCl, 10% (v/v) glycerol, 2.5 mM β-mercaptoethanol, and 20 mM imidazole), and then loaded into an XK column (GE Healthcare Life Sciences, Piscataway, NJ) and washed to base line on an AKTA purifier. PICK1 PDZ-QSAV was eluted from the nickel column using buffer A supplemented with 250 mM imidazole and analyzed by SDS-PAGE. The PICK1 PDZ-QSAV domain was then concentrated to 6 mg/ml and purified further on a Superdex 75 column equilibrated in buffer B (25 mM Tris-HCl pH 8.0, 250 mM NaCl, 5% (v/v) glycerol, and 2 mM DTT). PICK1 PDZ-QSAV eluted as a dimer and was approximately 95% pure based on SDS-PAGE. The PICK1 PDZ-QSAV was flash frozen and stored at –80°C.

His-MBP-PICK1 (1-355), a His-MBP-tagged PICK1 PDZ BAR domain construct lacking 59 residues of the C-terminal acidic region (CH1284), was expressed in the baculovirus vector expression system (BEVS). Baculovirus was generated using the pFast-Bac system in Sf9 cells and protein production was done in High Five Cells (Life Technologies) using MOI=1 and harvested 48 h post-transfection.

All purification steps were carried out at °C. The cell paste was suspended in cold lysis buffer (buffer A): 50 mM Tris-HCl pH 8.0, 1 M NaCl, 20 mM imidazole, 5% (v/v) glycerol, 0.75 mM DTT containing Roche complete EDTA free protease inhibitors, at a volume of

4 mL of lysis buffer/g of cell paste, and homogenized with a polytron (IKA T18 Ultra Turrax) followed by 2 passes with a microfluidizer. Lysate was clarified by centrifugation. Supernatant was batch-bound to Ni-NTA (Qiagen) (25 mL) and then resin collected by centrifugation before transferring to a column and washed with 14 column volumes (CV) of buffer A. The protein was eluted with buffer B (50 mM Tris-HCl pH 8.0, 500 mM NaCl, 250 mM imidazole, 5% (v/v) glycerol, 0.75 mM DTT, protease inhibitors. The CH1284 protein was purified further on a Superdex 200 size exclusion column (GE Healthcare 5.0 cm × 90 cm) using buffer C (50 mM Tris-HCl pH 8, 200 mM NaCl, 5% (v/v) glycerol, 2 mM DTT). The protein was evaluated for purity by analytical gel filtration (1 cm × 30 cm Superdex 200 GE Healthcare). It was flash frozen and stored at –80°C.
